# A Novel Mutant Allele of Pw1/Peg3 Does Not Affect Maternal Behavior or Nursing Behavior

**DOI:** 10.1371/journal.pgen.1006053

**Published:** 2016-05-17

**Authors:** Anne-Lyse Denizot, Vanessa Besson, Rosa Maria Correra, Alessia Mazzola, Izolina Lopes, Jean-Remy Courbard, Giovanna Marazzi, David A. Sassoon

**Affiliations:** Stem Cells and Regenerative Medicine, Institute of Cardiometabolism and Nutrition (ICAN), UMRS 1166 INSERM, University of Pierre and Marie Curie Paris VI, Paris, France; University of Pennsylvania, UNITED STATES

## Abstract

Parental imprinting is a mammalian-specific form of epigenetic regulation in which one allele of a gene is silenced depending on its parental origin. Parentally imprinted genes have been shown to play a role in growth, metabolism, cancer, and behavior. Although the molecular mechanisms underlying parental imprinting have been largely elucidated, the selective advantage of silencing one allele remains unclear. The mutant phenotype of the imprinted gene, *Pw1/Peg3*, provides a key example to illustrate the hypothesis on a coadaptation between mother and offspring, in which *Pw1/Peg3* is required for a set of essential maternal behaviors, such as nursing, nest building, and postnatal care. We have generated a novel *Pw1/Peg3* mutant allele that targets the last exon for the PW1 protein that contains >90% of the coding sequence resulting in a loss of *Pw1/Peg3* expression. In contrast to previous reports that have targeted upstream exons, we observe that maternal behavior and lactation are not disrupted upon loss of *Pw1/Peg3*. Both paternal and homozygous *Pw1/Peg3* mutant females nurse and feed their pups properly and no differences are detected in either oxytocin neuron number or oxytocin plasma levels. In addition, suckling capacities are normal in mutant pups. Consistent with previous reports, we observe a reduction of postnatal growth. These results support a general role for *Pw1*/*Peg3* in the regulation of body growth but not maternal care and lactation.

## Author Summary

Parental genomic imprinting is a mammalian-specific form of epigenetic control that regulates genes differently depending upon whether they are paternally or maternally inherited. The selective advantage of genomic imprinting is poorly understood and has been the subject of numerous theories. In the last several decades, mouse genetic studies have revealed that imprinted genes regulate embryonic and postnatal growth, metabolism, stem cells, neuronal functions, and most notably, behavior. The paternally expressed gene *Pw1/Peg3* was one of the first imprinted genes shown to influence maternal behaviors essential for pup survival and growth. Several key studies have demonstrated that *Pw1/Peg3* is required for proper nursing and milk ejection by the mother and suckling by the offspring. These previous observations have provided a strong support for the coadaptation theory of imprinting, which proposes that imprinted genes regulate the use of resources between mother and progeny to optimize their survival and future reproductive success. Here we describe that *Pw1/Peg3* mutant females exhibit intact maternal behaviors and do not display milk ejection defects. In addition, mutant pups are able to nurse properly.

## Introduction

Parental imprinting is a form of epigenetic regulation that results in an allele-specific expression of a gene according to its parental origin and is restricted to placental mammals [1–3]. Since 1991, about 100 parentally imprinted genes have been identified and mutant mice have been generated for many of these genes (reviewed in [4–6]). The selective advantages of parental imprinting remain unclear. Analyses of mice carrying mutations in parentally imprinted genes as well as genetic diseases corresponding to parentally imprinted genes in humans have shown that many of these genes play key roles in regulating body growth, metabolism, and adult behaviors [7–13].

*Pw1/Peg3* was identified initially in 1996 and is expressed primarily from the paternally inherited allele [14, 15]. PW1/PEG3 expression initiates upon gastrulation and persists at high levels in multiple embryonic tissues [14, 15]. Most postnatal and adult tissues exhibit restricted PW1/PEG3 expression in a small cell population with a notable exception of the brain, which maintains higher levels of expression in the neuronal lineage throughout adult life [10, 15, 16]. A constitutive *Pw1/Peg3* knockout mouse mutant was generated previously and analyses of the paternal mutants (*Pw1/Peg3*^*m+/p-*^) revealed perinatal growth retardation [10]. In addition, *Pw1/Peg3*^*m+/p-*^ females displayed impairments in nest building, pup retrieval, and decreased milk ejection leading to decreased offspring survival [10, 17, 18]. Li and colleagues [10] identified a decrease in oxytocin-expressing neurons as one primary mechanism underlying these maternal defects. Loss-of-function of another parentally imprinted gene, *Mest/Peg1*, also results in maternal behavior defects [7]. These observations, made more than 20 years ago, were viewed within the context of two prevailing theories regarding the role of parentally imprinted genes and their unique epigenetic control. The first theory, referred to as the ‘*parental conflict hypothesis*’ (also known as the ‘*kinship theory*’) proposes that the paternal contribution to the offspring drives embryonic and postnatal growth, whereas the maternal contribution limits growth of the offspring, yet promotes the conservation of maternal resources that would favor maternal survival [19]. The second proposal, referred to as the ‘*coadaptation theory*’, posits that these genes are critical for optimizing maternal-offspring survival [20]. These two theories are compatible in that the paternal drive of the offspring growth would be of a little value if the mother either did not survive pregnancy or failed to adequately care for her young, implying that a balance of these two directives is essential for survival and future reproductive success.

*Pw1/Peg3* was discovered in an effort to identify paternally expressed (Peg) or maternally expressed (Meg) genes in the mammalian genome [14] and independently in the same year from a screen for upstream regulators of stem cell specification [15]. Consistent with the latter strategy, it was found that PW1/PEG3 is expressed in adult somatic stem cells in a wide range of tissues including skeletal muscle, skin, gut, testis, and hematopoietic system, and central nervous system [16, 21, 22]. More recently, it has been demonstrated that PW1/PEG3 is required for stem cell competence in mesoangioblasts [23]. The regulatory role of PW1/PEG3 is likely to be complex as previous studies demonstrated that PW1/PEG3 participates in the two cell-stress signaling pathways—p53 and TNF/NFκB—leading to either cell death or survival, respectively [24, 25]. Pw1/Peg3 has also been shown to inhibit the Wnt signaling pathway by promoting β-catenin stabilization [26]. In addition, Pw1/Peg3 functions a transcription factor that regulates multiple genes involved in cellular metabolism [27]. Taken together, these results suggest that Pw1/Peg3 serves as a mediator of cell stress in adult stem/progenitor cells. Perera and colleagues recently generated a conditional *Pw1/Peg3* allele targeting the coding exon 6 [28]. They first induced a constitutive recombination of their mouse model and showed that paternal *Pw1/Peg3* deletion alone up-regulates the maternally expressed gene *Zim1* and results in postnatal growth defects. Interestingly, they reported *Pw1/Peg3* expression from the maternal allele in restricted regions of the neonatal and adult brain including the hypothalamus [28].

We report here the generation of a novel mutant allele for *Pw1/Peg3 (referred to henceforth as Pw1)*. In contrast to previously generated *Pw1* mutant mouse models, we targeted the last *Pw1* exon with loxP sites that contains >90% of the coding sequence and a putative transcription start site [15]. In order to compare our allele with previously reported results for constitutive loss-of-function models, we crossed our *Pw1* floxed mice with PGK-Cre mice to obtain offspring with germ-line mutation of *Pw1*. These mice were then used to establish a colony of constitutive mutant mice. Analyses of paternal, maternal, and homozygous mutant mice were carried out. We confirmed that our targeting strategy eliminated PW1 protein expression during development and in postnatal tissues including the hypothalamus, and as seen in previous studies, mutant mice are viable but display postnatal growth defects. Despite this phenotypic overlap with previous models in postnatal growth, a detailed analysis of paternal, maternal and homozygous *Pw1* mutant females showed that all aspects of maternal behavior (nest building, pup retrieval, crouching, pup sniffing, milk ejection) were indistinguishable from wild-type nulliparous and primiparous adult females. In addition, wild-type pups showed no difference in postnatal growth when nursed by wildtype or mutant mothers and no difference in mutant pup milk intake was observed between wildtype and mutant pups. Lastly, the number of oxytocin expressing neurons in oxytocin-producing hypothalamic nuclei were unaffected and the circulating levels of oxytocin were unchanged. Taken together, our data show that *Pw1* does not play a role in maternal behaviors, but does play a role in the regulation of postnatal growth. These findings may have implications with regard to prevailing theories on the evolutionary benefits of parental imprinting.

## Results

### Generation and characterization of a *Pw1* constitutive knockout mouse

**Fig 1:**
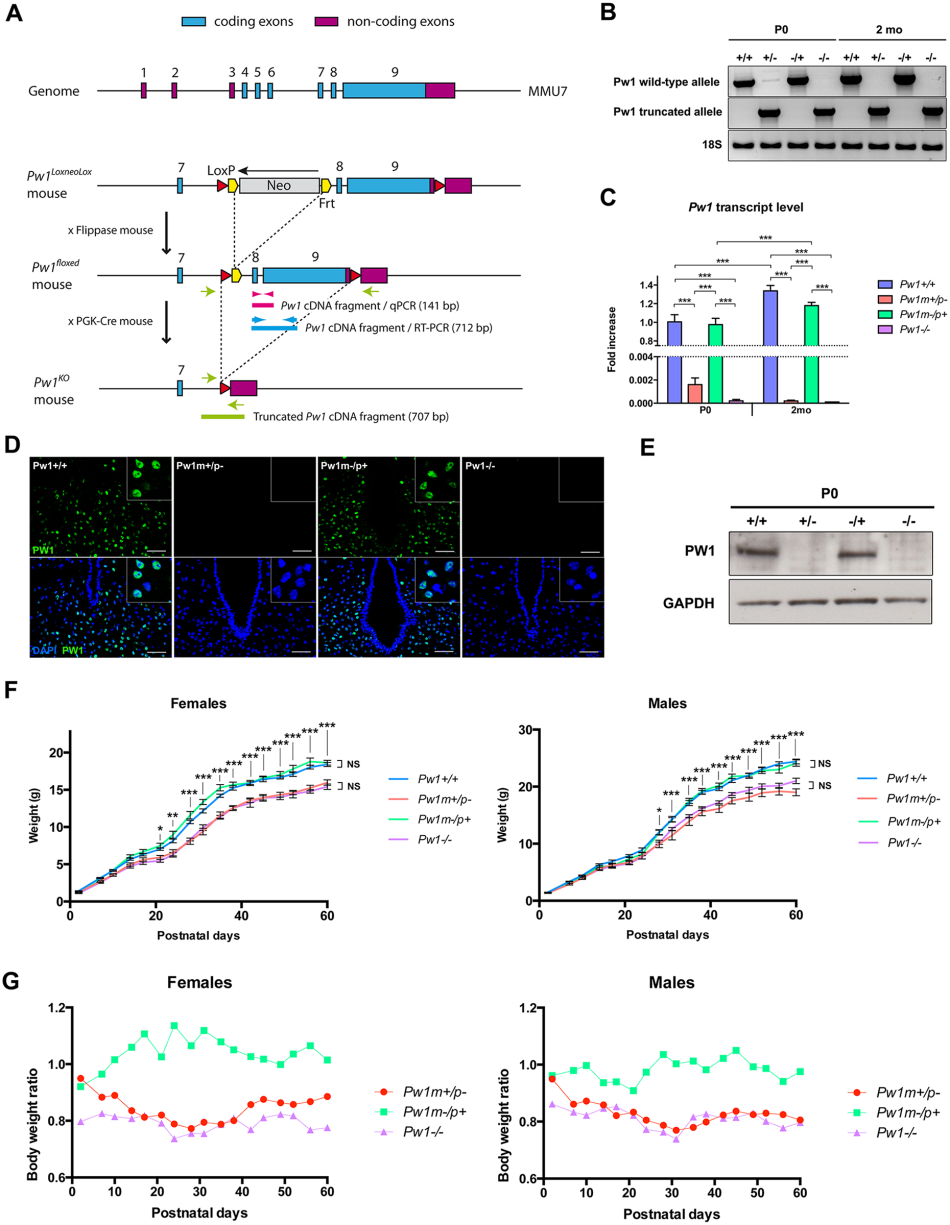
*Pw1* knockout strategy and characterization. **A.** *Pw1* knockout construct. Pink, blue, and green arrows-arrowheads correspond to location of *Pw1* primers. **B.** Expression levels of *Pw1* wildtype and *Pw1* truncated knockout alleles from semi-quantitative RT-PCR analysis in postnatal day 0 (P0) and 2 months old (2 mo) *Pw1*^*+/+*^ (+/+), *Pw1*^*m+/p-*^ (+/-), *Pw1*^*m-/p+*^ (-/+), and *Pw1*^*-/-*^ (-/-) brains (n = 3). **C.** Expression level of *Pw1* wild-type allele from real time PCR normalized to *Hprt1* gene (n = 3). **D.** PW1 immunofluorescence (green) on 3–4 months old postpartum female hypothalamus (retrochiasmatic area) (n≥4). Nuclei were counterstained by DAPI. Scale bar: 50μm. **E.** Western blot analysis showing levels of PW1 at P0 in *Pw1*^*+/+*^ (+/+), *Pw1*^*m+/p-*^ (+/-), *Pw1*^*m-/p+*^ (-/+), and *Pw1*^*-/-*^ (-/-) brains (n = 3). **F. Left panel:** Postnatal growth of *Pw1*^*+/+*^ (n = 26), *Pw1*^*m+/p-*^ (n = 12), *Pw1*^*m-/p+*^ (n = 5), and *Pw1*^*-/-*^ (n = 5) female mice. **Right panel:** Postnatal growth of *Pw1*^*+/+*^ (n = 18), *Pw1*^*m+/p-*^ (n = 10), *Pw1*^*m-/p+*^ (n = 9), and *Pw1*^*-/-*^ (n = 4) male mice. Paternal loss of *Pw1* leads to a reduced postnatal growth. **G.** Data shown in F presented additionally as percentage of *Pw1*^*+/+*^ littermates weight. In all graphs except panel G, values represent mean ± s.e.m. Statistical analysis was performed using two-way ANOVA test. *P<0.05, **P<0.01, and ***P<0.001. NS: non-significant.

In this study, we have generated a *Pw1* conditional knockout mouse targeting *Pw1* exons 8 and 9 (Fig 1A, S1 Fig). The neomycin ES-cell selection cassette was excised by crossing with a mouse line carrying flippase, which recognizes and recombines Frt sites [29] (Fig 1A). To generate a constitutive *Pw1* knockout mouse, we crossed the Pw1-floxed with a constitutive PGK-Cre mouse [30] (Fig 1A). The mutant mice were viable and survived to adult stages. As a first step to validate our constitutive *Pw1* knockout mouse model, we verified that paternal loss of *Pw1* leads to loss of PW1 protein during early embryonic development (S5G Fig). We next examined the *Pw1* transcript and protein levels in the whole brain. We detected a truncated *Pw1* transcript from the recombined paternal allele suggesting that the targeted allele underwent correct transcriptional regulation (Fig 1B). No expression from the maternal *Pw1* allele was detected in 2 months-old *Pw1*^*m+/p-*^ mutant brains (Fig 1B–1E). In contrast, we detected a low level of maternal *Pw1* transcription in *Pw1*^*m+/p-*^ P0 brains samples using semi-quantitative RT-PCR (Fig 1B).

### Paternal loss of *Pw1* leads to reduced postnatal growth

Given the shared phenotype reported for the previously generated *Pw1* mutant mice for reduced postnatal growth [10, 17, 28], we monitored postnatal growth of our *Pw1*^*m+/p-*^, *Pw1*^*m-/p+*^, and *Pw1*^*-/-*^ mice. We found that postnatal growth of *Pw1*^*m+/p-*^ and *Pw1*^*-/-*^ mice displayed a significant reduction by postnatal day 21 (Fig 1F and 1G), whereas maternal *Pw1* deletion had no detectable effect upon postnatal growth. After weaning, the growth defect observed in *Pw1*^*m+/p-*^ and *Pw1*^*-/-*^ mice persisted through adulthood with 15 to 20% weight reduction by 2 months of age in both sexes. While overall body sizes were decreased, the weight of *Pw1*^*m+/p-*^ and *Pw1*^*-/-*^ brains did not show any significant differences between genotypes. Since the body sizes of the mutant mice were smaller in both *Pw1*^*m+/p-*^ and *Pw1*^*-/-*^ mice, the brains were proportionally larger (S3 Fig). Furthermore, *Pw1*^*-/-*^ mice and *Pw1*^*m+/p-*^ mice reduction in body size were the same, therefore the *Pw1* maternal allele expression detected in *Pw1*^*m+/p-*^ newborn brains is not sufficient to rescue body growth. Prenatal analyses revealed a placental component to the postnatal growth restriction of *Pw1*^*m+/p-*^ pups (S2 Fig). E17.5 *Pw1*^*m+/p-*^ fetuses showed a decreased placenta weight compared to their wildtype littermates with no concomitant embryo weight effect (S2 Fig).

### Reproduction, litter size, and maternal behaviors are not affected in *Pw1* mutant mice

**Fig 2:**
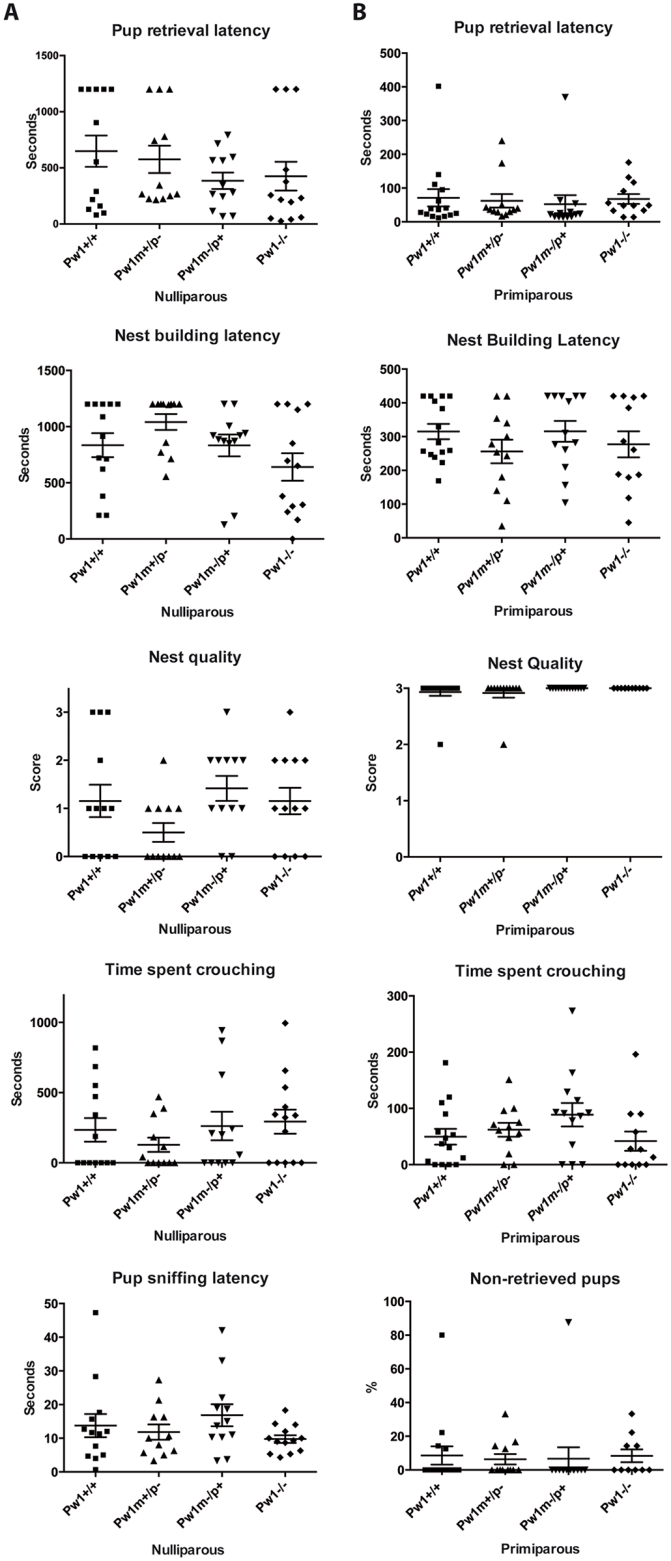
Maternal care is not impaired in *Pw1* mutant mice. **A.** Assessment of maternal behavior in 2 months old nulliparous (virgin) females (n≥12) using 3 foster pups of 1 to 3 days old chosen randomly. **B.** Assessment of maternal behavior in 3 to 4 months old primiparous females on the day of delivery (n≥12) using the female own litter. Nest quality is scored as followed: 0 = no nest building activity/no nest built; 1 = quick nest building activity, few nest materials/twigs have been retrieved; 2 = consequent nest building activity with some twigs remaining outside the nest. 3 = perfect nest without any twig left outside the nest. In all graphs, values represent mean ± s.e.m. Statistical analysis was performed using nonparametric one-way ANOVA (Kruskal-Wallis test). No significant differences were found between any of the four genotypes.

We monitored the matings of *Pw1*^*m+/p-*^, *Pw1*^*m-/p+*^, and/or *Pw1*^*-/-*^ mice and did not detect any impact on the percentage of post coitum pregnancies and all crosses carried out gave rise to the expected Mendelian ratios (Table 1). Importantly, mutant mothers appeared to properly care for their pups and litter sizes recorded at postnatal day 0 were similar in all cases with no significant differences in pup mortality indicating that the mutant mothers were not impaired in bringing their pups to weaning age (Table 1, S4 Fig). While statistically non-significant, we noted that pup mortality tended to be lower with *Pw1* mutant female mothers as compared to wild-type mothers; although whether this is due to pup genotype (*Pw1*^*+/+*^ and *Pw1*^*m-/p+*^) or female genotype remains unclear (S4 Fig). Given the previous reports that maternal behavior, and more specifically, that maternal care for pups is impaired in *Pw1* mutant female mice, we examined a range of maternal behaviors in our mutant allele. Maternal behaviors were assessed by measuring pup retrieval latency, nest building latency, nest quality, and the time spent crouching over the pups in nulliparous (virgin) and primiparous female mice. In addition, we measured the time nulliparous females took to acknowledge pup presence by recording pup sniffing latency, to establish a baseline between all genotypes. Among all the behaviors recorded, we did not detect any impairment in maternal care in nulliparous and primiparous *Pw1* mutant females (Fig 2A and 2B, S1 Movie). Even though *Pw1*^*m+/p-*^ nulliparous females appear to have a slightly increased nest building latency and a decreased nest quality, they behave similarly to wildtype upon primiparity. Importantly, no statistically significant differences were found between all four genotypes either in nulliparous and primiparous females (see supplementary statistical details). Additionally, the percentage of non-retrieved pups born to any group of primiparous mothers did not exceed 8.6% (Fig 2B). We note that nulliparous females did not show any significant differences in pup sniffing latency among all genotypes thereby allowing for a direct comparison of maternal behaviors for all genotypes examined (Fig 2A).

**Table 1:** Reproduction parameters of *Pw1* mutant mice are comparable to *Pw1*^*+/+*^ mice.

Cross		% Birth (plug efficiency)	Litter size *(Litter number)*	Mendelian inheritance			
Female genotype	Male genotype			*Pw1*^*+/+*^	*Pw1*^*-/+*^	*Pw1*^*-/-*^	*n pups*
***Pw1***^***+/+***^	***Pw1***^***+/+***^	73.8% ±18	8.1 ±0.5 *(14)*	100% ±0	0% ±0	0% ±0	*23*
***Pw1***^***+/+***^	***Pw1***^***m+/p-***^	69.1% ±7	8.6 ±0.4 *(9)*	46.1% ±6	53.9% ±6	0% ±0	*91*
***Pw1***^***m+/p-***^	***Pw1***^***+/+***^	80.0% ±19	8.0 ±0.3 *(20)*	54.3% ±4	45.7% ±4	0% ±0	*63*
***Pw1***^***m+/p-***^	***Pw1***^***m+/p-***^	62.7% ±8	7.9 ±1.0 *(7)*	24.5% ±7	49.2% ±6	26.3% ±7	*55*
***Pw1***^***+/+***^	***Pw1***^***-/-***^	69.4% ±3	8.1 ±0.4 *(10)*	0% ±0	100% ±0	0% ±0	*10*
***Pw1***^***-/-***^	***Pw1***^***+/+***^	61.7% ±24	7.7 ±0.3 *(15)*	0% ±0	100% ±0	0% ±0	*22*
***Pw1***^***m-/p+***^	***Pw1***^***+/+***^	62.5% ±19	8.5 ±0.4 *(13)*	ND	ND	ND	-
***Pw1***^***-/-***^	***Pw1***^***-/-***^	ND	ND	0% ±0	0% ±0	100% ±0	*14*

Values represent mean ± s.e.m. ND = not determined. Total litter number analyzed for litter size is indicated in brackets for each breeding type. Total pup number analyzed for Mendelian inheritance is indicated for each breeding type in the far right column.

### Oxytocin circulating level in females is not decreased upon *Pw1* deletion

**Fig 3:**
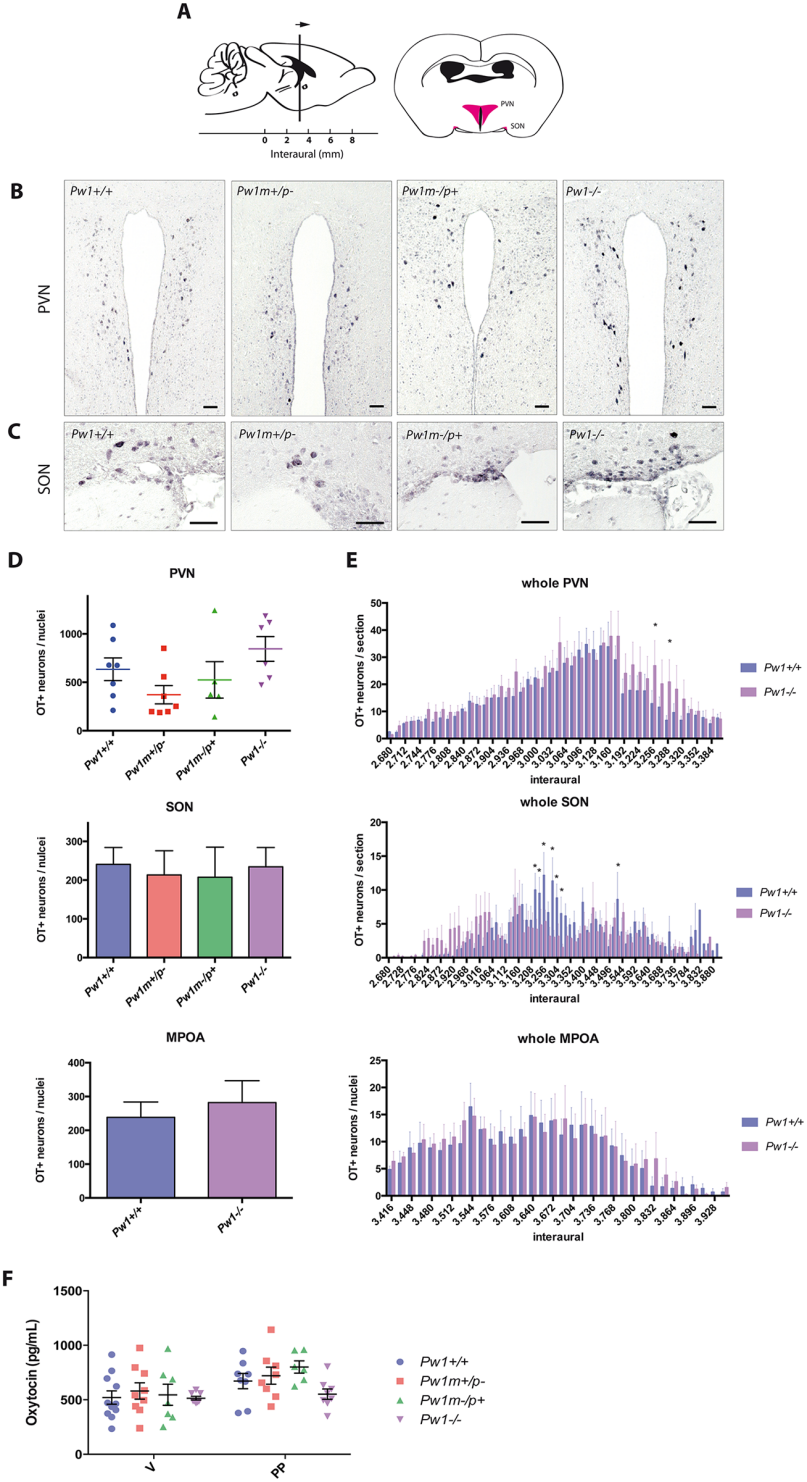
*Pw1* deletion does not result in significant decrease in oxytocin production and release. **A.** Left panel: schematic sagittal section of the adult mouse brain showing sectioning direction (arrow) on interaural coordinates. Right panel: schematic coronal section of the adult mouse brain showing the paraventricular nuclei (PVN) and the supraoptic nuclei (SON) in pink. **B-C.** Immunohistochemistry for oxytocin-expressing neurons in the PVN (B) and SON (C) of postpartum female brains (*Pw1*^*+/+*^, n = 7; *Pw1*^*m+/p-*^, n = 7; *Pw1*^*m-/p+*^, n = 5; *Pw1-/-*, n = 6). Scale bar: 50μm. **D.** Total number of oxytocin (OT) positive neurons per nuclei as stained as in Fig 3B and 3C (*Pw1*^*+/+*^, n = 7; *Pw1*^*m+/p-*^, n = 7; *Pw1*^*m-/p+*^, n = 5; *Pw1-/-*, n = 6). Bottom panel: total number of oxytocin (OT) positive neurons per medial preoptic area (MPOA) (*Pw1*^*+/+*^, n = 6; *Pw1-/-*, n = 6). No significant differences were found between all four genotypes. **E.** Number of oxytocin-positive neurons per section as stained as in Fig 3B and 3C for *Pw1*^*+/+*^ and *Pw1*^*-/-*^ postpartum female brains. **F.** Oxytocin plasma level in virgin (V) and postpartum (PP) females (V: *n* = 11, 9, 7, and 8; PP: n = 8, 8, 6, and 8; for *Pw1*^*+/+*^, *Pw1*^*m+/p-*^, *Pw1*^*m-/p+*^, *Pw1*^*-/-*^ females, respectively). *Pw1*^*-/-*^ postpartum females tend to have a lower oxytocin plasma level but this observation is not statistically significant. In all graphs, values represent mean ± s.e.m. Statistical analysis was performed using nonparametric one-way ANOVA (Kruskal-Wallis test) (Fig 3D), multiple t-tests (Fig 3E) or two-way ANOVA test (Fig 3F). *P<0.05, **P<0.01, and ***P<0.001. NS: non-significant.

Oxytocin is a peptide hormone synthesized in the hypothalamus. It has two different sites of release. In the brain parenchyma, oxytocin induces social bonding, notably maternal care [31–34]. Circulating oxytocin targets myoepithelial cells in the mammary gland to stimulate milk ejection and smooth muscle contraction in the uterus upon parturition [35]. Li and colleagues reported a decrease in the population of oxytocin-expressing neurons and suggested this was the most likely basis for the decrease in maternal care in *Pw1*^*m+/p-*^ females described in their study [10]. Consistent with a decrease in oxytocin expressing neurons, they also showed that *Pw1*^*m+/p-*^ females displayed a decreased capacity for milk ejection in nursing mutant female mice [10]. We therefore examined both oxytocin neuron number and circulating levels of oxytocin. We quantified the number of oxytocin-expressing neurons in the paraventricular nuclei and the supraoptic nuclei and found no differences between all four genotypes (Fig 3A–3E). We also quantified the number of oxytocin-expressing neurons in the medial preoptic area and found no differences between *Pw1*^*+/+*^ and *Pw1*^*-/-*^ females (Fig 3D and 3E). Consistent with these data, we found that oxytocin plasma levels in nulliparous females were comparable among all genotypes (Fig 3F). As expected, oxytocin plasma levels increased slightly upon parturition. Compared to wildtype, *Pw1*^*m+/p-*^ and *Pw1*^*m-/p+*^ mutant females, both nulliparous and primiparous, show no significant change in oxytocin systemic release (Fig 3F). It is noteworthy that levels of oxytocin were highly variable necessitating a large sample size. While our data reveal that loss of *Pw1* function does not result in a decrease in oxytocin levels, it is striking that the *Pw1*^*-/-*^ females display less variability in these levels indicating that there may be a more subtle role for PW1 in oxytocin regulation.

### Lactation and suckling in *Pw1* deficient mice is unchanged

**Fig 4:**
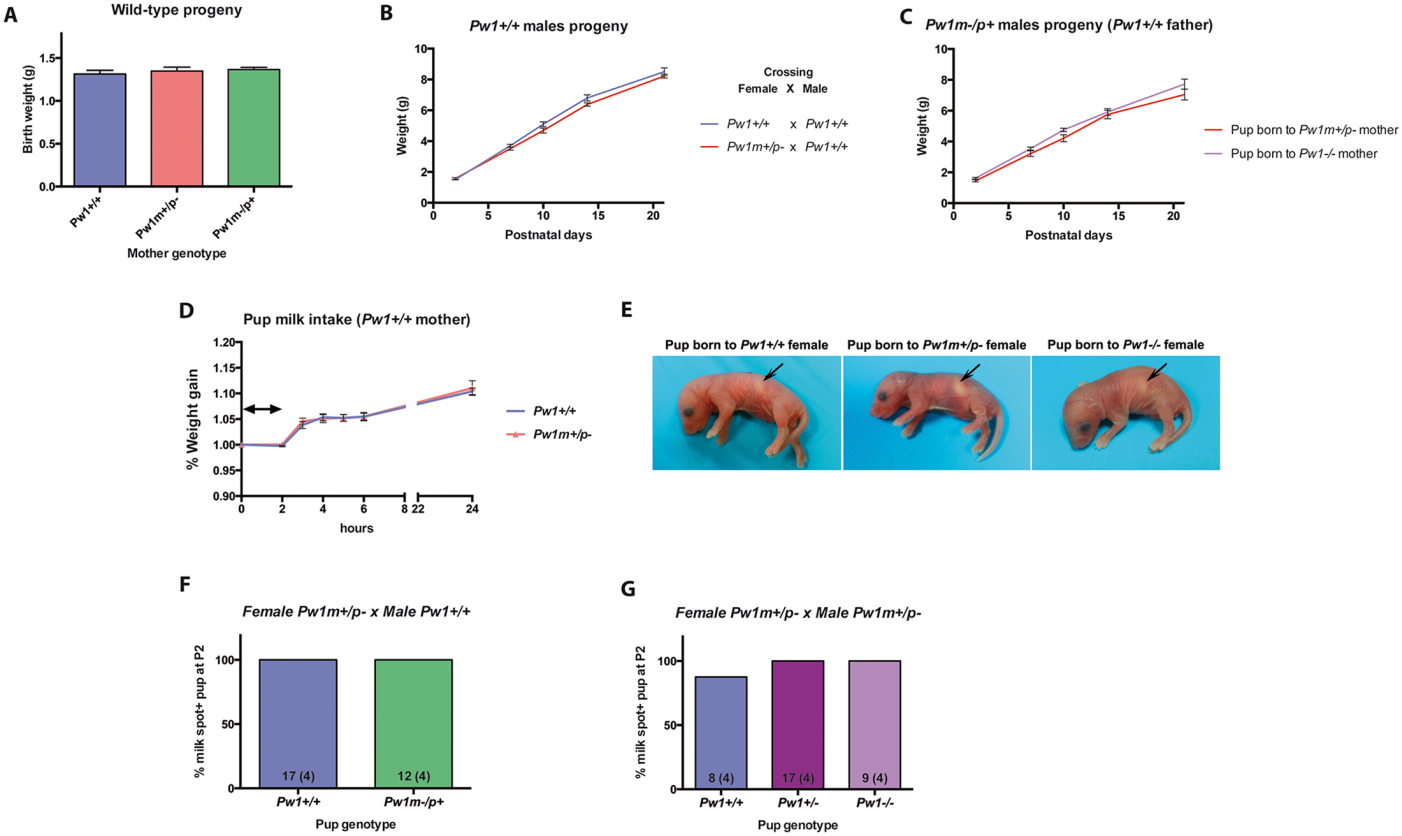
Lactation is not compromised in *Pw1* mutant mice. **A.** Birth weight of *Pw1*^*+/+*^ pups born to *Pw1*^*+/+*^, *Pw1*^*m+/p-*^, or *Pw1*^*m-/p+*^ mothers is unchanged (n = 7, 13, and 8 pups, respectively). **B.** Early postnatal growth of wild-type progeny of *Pw1*^*m+/p-*^ mothers is comparable to wild-type progeny of *Pw1*^*+/+*^ mothers. Weights were measured at postnatal days 2, 7, 10, 14, and 21, prior to weaning (n = 15, and n = 14 pups from at least 7 breedings *Pw1*^*+/+*^ x *Pw1*^*+/+*^, and 7 breedings *Pw1*^*m+/p-*^ x *Pw1*^*+/+*^, respectively). No significant differences were found. **C.** Early postnatal growth of *Pw1*^*m-/p+*^ progeny to *Pw1*^*m+/p-*^ and *Pw1*^*-/-*^ mothers crossed with a *Pw1*^*+/+*^ male are comparable. Weights have been measured at postnatal days 2, 7, 10, 14, and 21, prior to weaning (n = 9, n = 11, for breedings *Pw1*^*m+/p-*^ x *Pw1*^*+/+*^ and *Pw1*^*-/-*^ x *Pw1*^*+/+*^, respectively). No significant differences were found. **D.** Milk intake was assessed by measuring the gain of pup weight after a 2 hour starvation period at postnatal day 7. Milk intake of *Pw1*^*m+/p-*^ pups was similar to *Pw1*^*+/+*^ (*Pw1*^*+/+*^: n = 20 pups; *Pw1*^*m+/p-*^: n = 19 pups obtained from 5 independent breedings). The two-sided arrow indicates the 2 hour time-window when the pups were starved. **E.** Milk spot in day 0 pups (arrow). **F and G.** Percentage of postnatal day 2 pups showing a significant milk spot size from the following breedings: a female *Pw1*^*m+/p-*^ crossed with a male *Pw1*^*+/+*^ (F) and a female *Pw1*^*m+/p-*^ crossed with a male *Pw1*^*m+/p-*^ (G). The number of pups used is indicated on bars, with the number of independent breedings indicated in brackets. In all graphs, values represent mean ± s.e.m. Statistical analysis was performed using two-way ANOVA test.

Li and colleagues previously showed that *Pw1*^*m+/p-*^ mothers were deficient in milk ejection and suggested that this defect contributed to growth retardation of wild-type pup progeny [10]. In addition, Curley *et al* [36] and Kim *et al* [17] showed impaired suckling of *Pw1*^*m+/p-*^ pups born to wild-type mothers. However, we observed that wild-type progeny born to *Pw1*^*m+/p-*^ mothers did not show an impaired postnatal growth as compared to wild-type pups born to wild-type mothers (Fig 4A and 4B). Similarly, *Pw1*^*m-/p+*^ progeny born to *Pw1*^*m+/p-*^ or *Pw1*^*-/-*^ mothers did not display any postnatal growth differences (Fig 4C). Taken together, these results demonstrate that mutant mothers, carrying either a paternal mutant or homozygous mutant alleles for *Pw1*, do not show any defects in milk let-down or in milk ejection. Additionally, on the day of birth, pups born to mother specific genotype can show the same milk spot size (Fig 4E). By day 2, all the pups born to *Pw1*^*+/+*^, *Pw1*^*m+/p-*^, *Pw1*^*m-/p+*^, and *Pw1*^*-/-*^ mothers show a milk spot of a similar size (Fig 4F and 4G). As paternal loss of *Pw1* leads to growth reduction of pups that is detectable during weaning, we determined whether this was due to a decrease in milk intake in *Pw1*^*m+/p-*^ pups born to *Pw1*^*+/+*^ mothers by measuring weight gain of the pups. At postnatal day 7, *Pw1*^*m+/p-*^ pups displayed no detectable differences in milk intake as compared to wild-type pups (Fig 4D). We conclude that *Pw1*^*m+/p-*^ pups do not have any detectable suckling defects.

## Discussion

Three *Pw1* mutant mouse models have been previously and independently generated. In all three cases, mutant mice exhibited postnatal growth defects. While the initial studies of Kim *et al* [17] and Perera *et al* [28] did not examine behavioral phenotypes, Li *et al* [10] reported that the mothers did not properly care for their young and failed to eject milk correctly. Their study suggested that defects in postnatal growth were due to impairments in maternal function, since the wild-type pups cared by mutant mothers also showed a decreased postnatal growth prior to weaning [10]. However, unlike mutant pups, wild-type pups caught up to normal body size once weaned [10]. These data strongly supported a critical role for *Pw1* in maternal function. Moreover, Curley *et al* [36] and Kim *et al* [17] demonstrated that mutant pups have a decreased suckling ability, which leads to a decreased neonatal survival rate. Recently, Frey and Kim [37] have shown that their conditional mutant crossed onto a global Cre also results in milk let down as well as nest building defects in females. In their study, they describe several discrepancies with the study of Li *et al* [10] however their results confirmed a role for *Pw1* in maternal functions. Taken together, these studies showed that *Pw1* plays a role in maternal behavior, suckling, and body growth [10, 17, 36, 38] and provided supporting genetic evidence for the coadaptation theory of parentally imprinted gene function.

In the present study, we have generated a novel conditional *Pw1* allele and as a first step, we generated a constitutive loss-of-function line in order to compare the outcome with previously published results. While we confirm a postnatal growth defect in both paternal and homozygous *Pw1* mutant mice, we observe that all previously measured features of maternal behavior are indistinguishable from wild-type mice. How might we account for these phenotypic differences among different *Pw1* mutant models? In the course of our analyses, we first generated a conditional mouse line that retained the neomycin selection (*Pw1*^*loxneolox*^). We observed that the presence of the neomycin gene silenced PW1 expression as early as embryonic stage E12-13 (S5 Fig). A similar disruption of neighboring gene expression by neomycin insertion has been observed previously in multiple studies [39–41]. We therefore removed the neomycin cassette from our *Pw1* conditional mouse following a cross with the mouse carrying flippase [29], that lead to a complete restoration of normal *Pw1* expression during development and in the adult. A *Pw1* constitutive knock out mouse line was then obtained by crossing the *Pw1*^*floxed*^ mouse with a PGK-Cre mouse and bred onto a C57Bl/6J background [30]. While Li *et al* [10] inserted a promoterless *βgeo* cassette containing *LacZ* and *Neomycin* genes into *Pw1* coding exon 5, Kim *et al* [17] inserted a promoterless *LacZ* gene and a neomycin gene driven by the human β-actin promoter into *Pw1* intron 5 respectively, and Perera *et al* [28] deleted *Pw1* coding exon 6. In the first two mutants, the authors reported that a paternal deletion of *Pw1* led to a complete loss of PW1 protein expression. In contrast, Perera and colleagues [28] detected PW1 protein expression from both paternal and maternal alleles in the neonatal and adult brain. Notably, Perera *et al* [28] detected PW1 protein in the hypothalamus and choroid plexuses of *Pw1*^*m+/p-*^ neonatal and adult brains. Our strategy targeted coding exons 8 and 9 for *Pw1* [15] and we confirmed low levels of expression from the maternal *Pw1* allele in the neonatal brain. Consequently, studying all four genotypes (*Pw1*^*+/+*^, *Pw1*^*m+/p-*^, *Pw1*^*m-/p+*^, and *Pw1*^*-/-*^) was undertaken in this study. In addition, Relaix *et al* [15] showed that an alternative *Pw1* transcription start site is located at the 5'-end of *Pw1* coding exon 9, which is removed in our *Pw1* knockout mouse model. The *APeg3* gene is expressed as an antisens RNA in vasopressinergic magnocellular neurons of the hypothalamus [42, 43]. Our *Pw1* knock-out mouse construct is predicted to truncate *APeg3*, *h*owever, *APeg3* has only been described thus far to downregulate *Pw1* transcript and protein levels [44] thus no additional effects would be predicted although they cannot be ruled out. Taken together, it is possible that different construct strategies used for the generation of the published *Pw1* mutant mouse models and our model generated here may explain the lack of a maternal behavior and nutritional deficit in our model. Additionally, housing conditions can impact animal behavior. In the study by Li and colleagues, mice were housed in a reversed light cycle and experiments were performed during dark period [10] whereas our experiments on behavior were performed during the light cycle. Nonetheless, light and dark phase testing have been shown to score similar social behaviors [45]. We note however that pup retrieval and nest building latencies observed by Champagne *et al* [38] were obtained using C57Bl6 mouse strain in reversed light cycle method we do actually obtain very similar results. Lastly, variability between mouse strains may account for phenotype differences. While we used C57Bl/6J mice, Li *et al* [10] generated their *Pw1* mutant mice in 129Sv mouse strain [10]. Nonetheless, they later derived their mutant mouse model in a C57Bl/6J background and reproduced the maternal behavior phenotype [38]. The authors suggested that there was compensation over time since their original 129Sv *Pw1* mutant mice showed less maternal behavior defects after multiple generations [38]. We suggest therefore that the different targeting strategies used to generate the various *Pw1* mutant account for the differences we see as compared to the previously reported behavioral phenotypes [10, 17], Apart from the specific constructs used to generate *Pw1* mutant mice, experimental design can also impact phenotypic outcomes including breeding strategies. According to Curley, Broad, and Keverne [36, 46], *Pw1* acts synergistically at the maternal hypothalamus, the placenta, and the fetal hypothalamus to ensure reproductive success. *Pw1* expression in the placenta and fetal hypothalamus enhances maternal care and lactation, which suggests that offspring genotype affects mother phenotype. Curley *et al* [36] noted that "when the mutation was in the foetus, wild-type mothers ate less and failed to increase their food intake in the last week of pregnancy, suggesting an impairment of placental endocrine signals that are, in part, responsible for regulating maternal food intake." If such a generational impact is verified, the genotype of the offspring (n+1) is critical. In our behavioral analysis, females were bred to *Pw1*^*+/+*^ males such that offspring were either *Pw1*^*+/+*^ or *Pw1*^*m-/p+*^, which should leave PW1 expression unperturbed. Therefore we could compare all female genotypes together. Importantly, according to Curley and colleagues [36] the maternal weight gain of *Pw1*^*+/+*^ mothers during pregnancy carrying *Pw1*^*m+/p-*^ fetuses is decreased, to the same extent as *Pw1*^*m+/p-*^ mothers carrying *Pw1*^*+/+*^ pups. Accordingly, they suggested that *Pw1* functions are synchronized and optimized between mother and infant as a coadaptation phenomenon; *Pw1* expression in the fetus enhances *Pw1* functions in the mother. Whether the same defects are found regarding maternal behavior, nursing, thermogenesis in *Pw1*^*+/+*^ mothers carrying *Pw1*^*m+/p-*^ fetuses compared to *Pw1*^*m+/p-*^ mothers carrying *Pw1*^*+/+*^ pups remains to be determined. Additionnally, Curley *et al* [36] showed that wild-type pups born to *Pw1*^*m+/p-*^ mothers have a decreased birth weight and a reduced postnatal growth up to weaning. Using our *Pw1* mutant mouse model, we do not reproduce this result revealing that in our conditions the mother genotype does not impact pre- and postnatal growth of the pups. Moreover, we do not see any increase in perinatal death upon paternal and homozygous *Pw1* deletion in both mother and offspring.

To date, it remains unresolved as to how parental imprinting confers a selective advantage. A coadaptation theory was proposed by Wolf and Hager [47] relying on an evolutionary mathematical model and independently by Curley and Keverne [20, 36]. The latter group examined the existing *Pw1* mutant mouse and observed a suckling impairment in mutant pups. Broad and Keverne [20, 46] also provided evidence that *Pw1* regulates the expression of genes in the placenta, as well as in the fetal and maternal hypothalamus. Based on these observations, they suggested that *Pw1* is a key player governing care and nursing by the mother and resource consumption (suckling) by the offspring with subsequent effects upon postnatal growth, and future maternal care in pups adult life. Along with *Pw1*, *Mest/Peg1* was known to promote maternal bonding [7] and *Grb10* was recently shown to control nutritional resources both in mother and infant [48, 49]. A master-regulator role for *these imprinted genes* in regulating placental-fetal-maternal interactions is attractive since parental imprinting of these specific genes is unique to placental mammals.

We note that growth restriction is a central defect that is common to all *Pw1* mutant mouse models previously established as well [10, 17, 28] as the novel mutant allele reported in this study. Even though we observe that *Pw1*^*m+/p-*^ and *Pw1*^*-/-*^ pups are significantly smaller around postnatal day 21, they tend to have a decreased weight by postnatal day 2. Indeed Li and colleagues [10] demonstrated a growth restriction starting at E17.5. Thus, promoting growth may be one of the most important and most robust function regulated by *Pw1*. However, even in this regard our results differ with the observations reported [17, 36], since we observe that *Pw1*^*m+/p-*^ pups do not exhibit any suckling defect suggesting that growth restriction is completely intrinsic to *Pw1*^*m+/p-*^ and *Pw1*^*-/-*^ mice with a putative placental component (S2 Fig).

We did not observe *Pw1* maternal allele expression upon deletion of the *Pw1* paternal allele in the adult brain in our model. Thus, there is no loss of imprinting in the conditions tested. However, we detected *Pw1* maternal allele transcription at postnatal day 0 in *Pw1*^*m+/p-*^ brains. Whether this weak expression of the normally silenced copy is due to *Pw1* paternal deletion or reflects a normal relaxation of imprinting at birth remains to be resolved. Nonetheless, our observations are consistent with recent findings of Perera *et al* [28] in the neonatal brain. Our results indicate that maternal-allele driven *Pw1* expression is not sufficient to rescue postnatal growth defects. While the role, if any, of the maternal allele remains to be elucidated, we note that we detected transcription from the truncated paternal allele in *Pw1*^*m+/p-*^ and *Pw1*^*-/-*^ brains at high levels. As our PW1 antibody was generated to the coding domain present in the recombined 9^th^ exon [15], we cannot determine if a truncated PW1 protein is generated however it would represent a small portion of the entire PW1 protein. In addition, Ye and colleagues previously showed that PW1 is a transcriptional repressor of *Zim1 [50]*. Upon paternal loss of *Pw1*, they detected an increase in *Zim1* transcript level and validated PW1 binding to *Zim1* gene by ChIP analyses in the neonatal brain. However, in the present study we did not see any change in *Zim1* expression in *Pw1* knockout postnatal day 0 brains (S6A, S6B and S5B Figs).

In conclusion, the results presented here using a novel *Pw1* knockout mouse model demonstrate that *Pw1* is not essential for the maternal behaviors tested, milk letdown, or pup suckling ability, but rather *Pw1* primarily regulates intrinsic postnatal growth. Radford and colleagues [51] showed that *Pw1* is a stress-response gene during embryonic and fetal development. Specifically, upon maternal nutritional restriction, PW1 expression increases in the brain and liver of fetuses as a compensatory mechanism to ensure fetal growth. This stress response in the whole brain is consistent to our previous studies showing that PW1 participates in cell stress-responses leading to cellular growth arrest and cell death [24, 25]. More recently, we showed that PW1 expression marks stem/progenitor cells from all tissues examined to date including muscle resident stem cells, the hematopoietic system, brain, and skin [16] and is essential for proper somatic stem cell function [23]. Thus, *Pw1* may act as a coordinator to modulate growth cues to the whole body through direct action upon stem cells that underlie tissue growth representing an adaption at the organismal level.

## Materials and Methods

### Mice

A *Pw1* conditional knockout mouse line was generated at EMBL (Italy) by inserting two LoxP sites flanking *Pw1* exons 8 and 9 (Fig 1A, S1 Fig) (DNA source: pVBFRTCKR05-Peg3 20901bp). Four out of 600 clones were efficiently targeted using homologous recombination in an XY 129SvC57Bl6 hybrid ES cell line. The four positive clones were injected into C57BL/6 blastocysts and reimplanted into CD1 foster mothers. Karyotyping and Southern analyses were performed at EMBL. Out of the four resulting male chimeras, one founder was validated for further analyses. The Frt site flanked neomycin ES-cell selection cassette was excised by crossing with a mouse carrying flippase [29]. A constitutive *Pw1* knockout mouse model was subsequently obtained by crossing the *Pw1* floxed mouse with a constitutive PGK-Cre mouse [30] and expanded onto a C57Bl/6J background for at least 10 backcrosses prior to analysis (Elevage Janvier). The genotypes tested in this study were *Pw1*^*+/+*^*(wildtype)*, *Pw1*^*p-/m+*^*(paternal deletion)*, *Pw1*^*p+/m-*^ (*maternal deletion*) and *Pw1*^*-/-*^ (*homozygous deletion*) mice.

### Ethics statement

All work with mice was carried out in adherence to French government and European guidelines.

### RNA extraction, RT-PCR and RT-qPCR

RNA extracts were prepared using RNeasy Mini Kit (Qiagen) according to manufacturer's guidelines. RNA was treated with RNase-free DNase I (Qiagen) following the manufacturer's protocol. cDNAs were synthesized using random hexamers (SuperScript First-Strand Synthesis System; Life Technologies). Semi-quantitative polymerase chain reactions were carried out on a ProFlex PCR System. Cycling conditions were as follows: 95°C for 5 min followed by 30 (*18S*) to 34 (*Pw1*) cycles of amplification (95°C for 30 s, 56°C (truncated *Pw1*) 57°C (wild-type *Pw1*) or 60°C (*18S*) for 30 s and 72°C for 1 min), followed by a final incubation at 72°C for 10 min. Quantitative polymerase chain reaction was performed on a LightCycler 480 (Roche) using SYBR green (Thermo Fisher Scientific). Cycling conditions were as followed: 95°C for 5 min followed by 42 cycles of amplification (95°C for 15 s, 60°C for 15 s and 72°C for 20 s), then 95°C for 5 s followed by a final incubation at 65°C for 1 min.

Primers sequences used were: *Pw1* wild-type allele (semi-quantitative PCR) FWD 5'-AAGGCCACTCATCGAGGTCCAAGAGAACTGCC-3' and REV 5'-CCACATTCCTTACACTCAAAGC-3'; *Pw1* knockout allele FWD 5'-ACATGCCTGGAACTCCAGTGC-3' and REV 5'-ACCTTCACAGGACTATCTAAGAGGTAGGGG-3'; *18S* FWD 5'-CGGCTACCACATCCAAGGAA-3' and REV 5'-TATACGCTATTGGAGCTGGAA-3'; *Pw1* wild-type allele (qPCR) FWD 5'-TGGGAGTCCAGCTTGCCGAAGA-3' and REV 5'-CCACGCCTGTGGGATGGCTTT-3'; *Hprt1* FWD 5'-AGGGCATATCCAACAACAAACTT-3' and REV 5'-GTTAAGCAGTACAGCCCCAAA-3'. For real time PCR, levels of *Pw1* expression were normalized to *Hprt1* gene expression.

### Western analyses

Freshly isolated brains were homogenized in lysis buffer (150mM NaCl, 50mM Hepes pH7.6, 1% NP-40, 0,5% sodium deoxycholate, 5mM EDTA) supplemented with 1mM PMSF, Complete (Roche), 20mM NaF, 10mM b-glycerophosphate, 5mM Na-pyrophosphate, and 1mM Na-orthovanadate. Equal amounts of protein were separated by electrophoresis (Novex NuPAGE Bis-Tris protein gel 4–12% or 5% home-made Bis-Tris gel) and transferred to a PVDF membrane in 20% methanol transfer buffer. Membranes were probed with polyclonal rabbit antibodies to PW1 (rabbit, 1:10,000) (Relaix et al., 1996) and GAPDH (Abcam). Antibody binding was visualized using horse-radish peroxidase (HRP)-conjugated species-specific secondary antibodies (Jackson ImmunoResearch) followed by enhanced chemiluminescence (Pierce).

### Histological analyses

For immunofluorescence and immunohistochemistry experiments, animals were deeply anaesthetized and transcardially perfused with 4% paraformaldehyde (PFA), pH 7.4. Brains were post-fixed 18 hours at 4°C, cryoprotected overnight in 20% sucrose in PBS at 4°C, and snap frozen in isopentane at -50°C. Coronal cryosections (8μm) were processed for immunostaining. Permeabilization was performed in PBST (PBS, 0,1% Triton X-100) for 10 minutes. For PW1 staining alone, antigen retrieval was carried out in 0.01M citric acid, pH6.0, with two consecutive incubations of 5 minutes at 95°C. Sections were blocked by incubation for 1 hour in PBS supplemented with 4% IgG-free BSA (Jackson ImmunoResearch). Primary antibodies used were: PW1 (rabbit, 1:3,000) (Relaix et al., 1996), OXYTOCIN (rabbit, Abcam, 1:10,000). For immunofluorescence, antibody binding was visualized using rabbit-specific secondary antibody coupled to Alexa Fluor 488 (Life Technologies). Nuclei were counterstained with DAPI (Sigma). For immunohistochemistry, antibody binding was reacted using rabbit-specific secondary antibody coupled to biotin followed by streptavidine coupled to HRP (Jackson ImmunoResearch). The DAB enzymatic reaction was carried out using nickel enhancement according to manufacturer's instructions (VECTOR Laboratories). Oxytocin-positive neurons were quantified blind to genotype in the paraventricular nuclei by counting every other section from interaural coordinate 2.680 to 3.400 (from 2.680 to 3.900 for the supraoptic nuclei; from 3.416 to 3.944 for the medial preoptic area). Images were acquired using a Leica DM fluorescence and light microscope or Leica SPE confocal microscope.

### Maternal and reproduction behavior analyses

Maternal behavior was assessed based upon previously established protocols (Li *et al* and Champagne *et al*) [10, 38]. Observations were performed during the light period of a non-reversed light cycle cage facility. Females were accustomed to the experimental room at least 30 minutes prior testing. In their home-cage, each female was individually monitored for pup retrieval latency, time crouching over the whole pup set, nest quality and nest building latency. Pup retrieval was scored as the transfer of a pup into the nest. Nest quality was scored from 0 (absence of nest building activity) to 3 (perfectly built nest with no remaining nest material outside the nest). Accordingly, all cages were enriched with Cell Sizzle (SAFE) nest material for housing and maternal behavioral analyses. Observations and records were performed blind to genotype. The mean of each pup sniffing and retrieving latencies was used for both nulliparous and primiparous females.

Nulliparous (virgin) two months-old females were briefly removed from the test cage. Three newborn pups (0–3 days) were randomly chosen and placed interspaced in the test cage at the opposite side of the nest. In order to assess nest quality, the nest was gently disturbed by placing some nest material far from the nest. Nulliparous females were then transferred back to the test cage. Pup sniffing latency and maternal behaviors were recorded for 20 minutes. Nest quality was scored at the end of the test period. For primiparous females, three to four months old primary timed-pregnant females were isolated at gestation day 17 into a new cage with fresh nest material. On the day of birth (2-6pm), nest quality was scored before removing the female from the cage. All pups were placed interspaced at the opposite side of the nest before disturbing the nest gently. Upon retransfer of the female back to her pups, maternal behavior was monitored and recorded for 7 minutes. Behavioral analysis was carried out as described above except that the female was tested with her own full litter. A GoPro Hero3+ camera was fixed on top of the cage in order to better track maternal behavior. In addition, the number of dead pups (excluded from the test) and litter size were recorded.

Controlled matings were performed to assess reproductive competence. Around 5-6pm, two females were introduced to a male overnight. The following morning (9-10pm), females were checked for vaginal plug and separated from the male accordingly. Plugged females were monitored for gestation up to 17 days post-coitum. For each cross, at least three different males and a dozen of females were used for a total of 15 plugs analyzed. For this study, all animals were 2 to 6 months old.

### Milk intake

Pup milk intake ability was measured to assess suckling ability in postnatal day 7 wild-type and paternal mutant pups born to wild-type mothers in order to avoid potential contribution of the maternal genotype to suckling behavior. Pups were placed into a warm incubator for 2 hours allowing for the weight to stabilize followed by reintroduction to the mother. Individual pup weights were recorded hourly for 4 hours and again at 24 hours.

### Oxytocin measurements

Blood was collected from the tail of 3 months old nulliparous and 3–4 months old primiparous females using kalium-EDTA coated tubes (Sarstedt). Plasma were isolated according to manufacturer's instructions and stored at -80°C. Oxytocin plasma levels were measured using the Oxytocin ELISA kit (Enzo Life Sciences), following the manufacturer's protocol. Optical densities were measured on a FlexStation 3 (Molecular Devices) at 405nm, with correction at 580nm.

### Statistical analysis

All statistical analyses were performed using GraphPad Prism software, version 6.0. Tests carried out are mentioned in figure legends; they include one- or two-way ANOVA tests. Data are presented as the mean ± standard error of the mean (s.e.m.) *P<0.05, **P<0.01 and ***P<0.001. The number of animals used for each experimental condition (n) is indicated in the figure legends or in the above methods.

## Supporting Information

S1 Movie Maternal behavior of primiparous *Pw1*^*+/+*^, *Pw1*^*m+/p-*^, *Pw1*^*m-/p+*^ and *Pw1*^*-/-*^ females. One representative female per genotype is shown. Video has been provided accelerated. Normal total test duration is 7 minutes.
(MP4)

S1 Fig Southern Blot performed on recombined ES clones to confirm the correct insertion and orientation of the *Pw1* KO construct within the *Pw1* genomic locus. **A**. Scheme representing probes 1 and 2 used for the Southern Blot analyses. Black crosses indicate recombination sites. Proper insertion is predicted to generate distinct genomic fragments due to the presence of BglII (Bgl2) and NsiI restriction sites. Specifically, a 5,6 Kb and a 8,8 Kb fragment are detected with probes 1 and 2 respectively in the recombined (KO) allele whereas a 11,2 Kb and a 13,3 Kb fragment are predicted for the wildtype (WT) allele. **B**. Prior to Southern on the ES clones, probes 1 and 2 were tested in duplicate by Southern on C57Bl6 genomic DNA digested by BglII and NsiI respectively. **C-D**. Southern Blot on ES cells clones using probe 1 (C) and probe 2 (D). Out of ~600 Neomycin-selected-ES cells clones, 14 clones were identified by probes 1 and 2. Out of those 14, four clones (*) were finally selected for the blastocysts injection.
(TIF)

S2 Fig Paternal loss of *Pw1* affects placenta weight at fetal stage E17.5. **A**. Weight of *Pw1*^*+/+*^ compared to *Pw1*^*m+/p-*^ E17.5 littermate embryos. **B**. Weight of *Pw1*^*+/+*^ compared to *Pw1*^*m+/p-*^ E17.5 littermate embryos' placenta. **Panel A. Pup retrieval latency:** Values were not all normally distributed (P ≥ 0.0041, D'Agostino and Pearson omnibus normality test). Using Kruskal-Wallis test (P = 0.4171) no significant differences were found between all four genotypes. **Nest building latency:** Values were not all normally distributed (P ≥ 0.0383, D'Agostino and Pearson omnibus normality test). Using Kruskal-Wallis test (P = 0.1032) no significant differences were found between all four genotypes. **Nest quality:** Values were all normally distributed (P > 0.2142, D'Agostino and Pearson omnibus normality test). Using ordinary one-way ANOVA (F_3,46_ = 1.954; P = 0.1341) no significant differences were found between all four genotypes. **Time spent crouching:** Values were all normally distributed (P > 0.1567, D'Agostino and Pearson omnibus normality test). Using ordinary one-way ANOVA (F_3,46_ = 0.7315; P = 0.5385) no significant differences were found between all four genotypes. **Pup sniffing latency:** Values were not all normally distributed (P ≥ 0.0011, D'Agostino and Pearson omnibus normality test). Using Kruskal-Wallis test (P = 0.3885) no significant differences were found between all four genotypes. **Panel B: Pup retrieval latency:** Values were not all normally distributed (P < 0.0001, D'Agostino and Pearson omnibus normality test). Using Kruskal-Wallis test (P = 0.1346) no significant differences were found between all four genotypes. **Nest building latency:** Values were all normally distributed (P > 0.5123, D'Agostino and Pearson omnibus normality test). Using ordinary one-way ANOVA (F_3,48_ = 0.8627; P = 0.4669) no significant differences were found between all four genotypes. **Nest quality:** Values were not all normally distributed (P < 0.0001, D'Agostino and Pearson omnibus normality test). Using Kruskal-Wallis test (P = 0.5854) no significant differences were found between all four genotypes. **Time spent crouching:** Values were not all normally distributed (P ≥ 0.0027, D'Agostino and Pearson omnibus normality test). Using Kruskal-Wallis test (P = 0.1856) no significant differences were found between all four genotypes. **Non-retrieved pups:** Values were not all normally distributed (P < 0.0001, D'Agostino and Pearson omnibus normality test). Using Kruskal-Wallis test (P = 0.4047) no significant differences were found between all four genotypes. No outliers were removed prior analyses. Alpha = 0.05 for all tests (D'Agostino and Pearson omnibus and Kolmogorov-Smirnov tests: if P<0.05 data do not pass the normality test).
(TIF)

S3 Fig *Pw1* mutant and *Pw1*^*+/+*^ brains show the same weight and size with respect to the body weight and size, respectively. **A**. Brain weights of 2 months old *Pw1*^*+/+*^, *Pw1*^*m+/p-*^, *Pw1*^*m-/p+*^ and *Pw1*^*-/-*^ males. **B**. Representative picture of 2 months old *Pw1*^*+/+*^ and *Pw1*^*-/-*^ brains. **C.** Brain over body weights ratio of 2 months old *Pw1*^*+/+*^, *Pw1*^*m+/p-*^, *Pw1*^*m-/p+*^ and *Pw1*^*-/-*^ males. In all graphs, values represent mean ± s.e.m. Statistical analysis was performed using one-way ANOVA (n≥4). *P<0.05, **P<0.01 and ***P<0.001. **D. Upper panel—PVN.** All values were normally distributed (P > 0.1000, Kolmogorov-Smirnov test). Using one-way ANOVA (F_3,21_ = 2.468; P = 0.0902) no significant differences were found between all four genotypes. **Middle panel—SON.** All values were normally distributed (P > 0.0848, Kolmogorov-Smirnov test). Using one-way ANOVA (F_3,20_ = 0.07157; P = 0.9745) no significant differences were found between all four genotypes. **Bottom panel—MPOA**. All values were normally distributed (P > 0.1000, Kolmogorov-Smirnov test). Using unpaired t test no significant differences were found between the two genotypes (P = 05924). **F.** Using two-way ANOVA (Interaction: F_3,57_ = 0.7796; P = 0.5102) no significant differences were found.
(TIF)

S4 Fig Progenies of *Pw1* mutant mothers do not show increased death rate within the first 12 hours of life. Values represent mean ± s.e.m. Statistical analysis was performed using one-way ANOVA Kruskal-Wallis test (n≥12 litters).
(TIF)

S5 Fig Neomycin insertion downregulates *Pw1* expression. **A-C.** Postnatal day 0 brains were analyzed for *Pw1* (**A**), *Zim1* (**B**), and *Usp29* (**C**) expression levels by RT-qPCR. Values represent fold increase ± s.e.m. normalized to *Hprt1* expression level. Statistical analysis was performed using one-way ANOVA Kruskal-Wallis test (n = 3). *P<0.05, **P<0.01 and ***P<0.001. **D-F.** Immunostaining for PW1 on E11.5, E14.5, and E16.5 *Pw1*^*+/+*^ and *Pw1*^*+/loxneolox*^ embryos (8μm thick sections). **D**. Umbilical cord. **E**. Brain. **F**. Abdominal wall. In *Pw1*^*+/loxneolox*^ embryos, neomycin insertion irreversibly shuts down PW1 expression between E11.5 and E14.5. **G**. Immunostaining for PW1 on E11.5 *Pw1*^*+/+*^ and *Pw1*^*m+/p-*^ embryos (8μm thick sections). As expected, paternal loss of *Pw1* abrogates PW1 expression. Here is shown the umbilical cord, a strong site of PW1 expression at E11.5.
(TIF)

S6 Fig *Zim1*, *Usp29*, and *APeg3* transcript levels do not change upon loss of *Pw1*. **A.** *Zim1* real time RT-PCR on postnatal day 0 brains (n = 3 per genotype). Gene expression levels are normalized using *Hprt1* gene expression. **B**. *Zim1* and *Usp29 transcript levels analyzed by* semi-quantitative RT-PCR on postnatal day 0 brains (n = 3 per genotype). **C**. *APeg3* transcript level analysis on postnatal day 0 brains using First-Strand RT-PCR. Each well corresponds to a different sample.
(TIF)

## References

[1] McGrath J, Solter D. Completion of mouse embryogenesis requires both the maternal and paternal genomes. Cell. 1984;37(1):179–83. Epub 1984/05/01. 0092-8674(84)90313-1 [pii]. . 6722870 10.1016/0092-8674(84)90313-1

[2] Surani MA, Barton SC, Norris ML. Development of reconstituted mouse eggs suggests imprinting of the genome during gametogenesis. Nature. 1984;308(5959):548–50. Epub 1984/04/05. . 6709062 10.1038/308548a0

[3] Cattanach BM, Kirk M. Differential activity of maternally and paternally derived chromosome regions in mice. Nature. 1985;315(6019):496–8. Epub 1985/06/06. . 4000278 10.1038/315496a0

[4] Ferguson-Smith AC. Genomic imprinting: the emergence of an epigenetic paradigm. Nat Rev Genet. 2011;12(8):565–75. Epub 2011/07/19. nrg3032 [pii] 10.1038/nrg3032 . 21765458

[5] Plasschaert RN, Bartolomei MS. Genomic imprinting in development, growth, behavior and stem cells. Development. 2014;141(9):1805–13. Epub 2014/04/24. 141/9/1805 [pii] 10.1242/dev.101428 24757003 PMC3994769

[6] Peters J. The role of genomic imprinting in biology and disease: an expanding view. Nat Rev Genet. 2014;15(8):517–30. Epub 2014/06/25. nrg3766 [pii] 10.1038/nrg3766 . 24958438

[7] Lefebvre L, Viville S, Barton SC, Ishino F, Keverne EB, Surani MA. Abnormal maternal behaviour and growth retardation associated with loss of the imprinted gene Mest. Nat Genet. 1998;20(2):163–9. Epub 1998/10/15. 10.1038/2464 . 9771709

[8] Varrault A, Gueydan C, Delalbre A, Bellmann A, Houssami S, Aknin C, et al Zac1 regulates an imprinted gene network critically involved in the control of embryonic growth. Dev Cell. 2006;11(5):711–22. Epub 2006/11/07. S1534-5807(06)00396-0 [pii] 10.1016/j.devcel.2006.09.003 . 17084362

[9] Itier JM, Tremp GL, Leonard JF, Multon MC, Ret G, Schweighoffer F, et al Imprinted gene in postnatal growth role. Nature. 1998;393(6681):125–6. Epub 1998/05/29. 10.1038/30120 . 9603515

[10] Li L, Keverne EB, Aparicio SA, Ishino F, Barton SC, Surani MA. Regulation of maternal behavior and offspring growth by paternally expressed Peg3. Science. 1999;284(5412):330–3. Epub 1999/04/09. . 10195900 10.1126/science.284.5412.330

[11] Garfield AS, Cowley M, Smith FM, Moorwood K, Stewart-Cox JE, Gilroy K, et al Distinct physiological and behavioural functions for parental alleles of imprinted Grb10. Nature. 2011;469(7331):534–8. Epub 2011/01/29. nature09651 [pii] 10.1038/nature09651 21270893 PMC3031026

[12] Curley JP, Pinnock SB, Dickson SL, Thresher R, Miyoshi N, Surani MA, et al Increased body fat in mice with a targeted mutation of the paternally expressed imprinted gene Peg3. FASEB J. 2005;19(10):1302–4. Epub 2005/06/02. 04-3216fje [pii] 10.1096/fj.04-3216fje . 15928196

[13] Charalambous M, Ferron SR, da Rocha ST, Murray AJ, Rowland T, Ito M, et al Imprinted gene dosage is critical for the transition to independent life. Cell Metab. 2012;15(2):209–21. Epub 2012/02/14. S1550-4131(12)00010-1 [pii] 10.1016/j.cmet.2012.01.006 22326222 PMC3314949

[14] Kuroiwa Y, Kaneko-Ishino T, Kagitani F, Kohda T, Li LL, Tada M, et al Peg3 imprinted gene on proximal chromosome 7 encodes for a zinc finger protein. Nat Genet. 1996;12(2):186–90. Epub 1996/02/01. 10.1038/ng0296-186 . 8563758

[15] Relaix F, Weng X, Marazzi G, Yang E, Copeland N, Jenkins N, et al Pw1, a novel zinc finger gene implicated in the myogenic and neuronal lineages. Dev Biol. 1996;177(2):383–96. Epub 1996/08/01. S0012-1606(96)90172-4 [pii] 10.1006/dbio.1996.0172 . 8806818

[16] Besson V, Smeriglio P, Wegener A, Relaix F, Nait Oumesmar B, Sassoon DA, et al PW1 gene/paternally expressed gene 3 (PW1/Peg3) identifies multiple adult stem and progenitor cell populations. Proc Natl Acad Sci U S A. 2011;108(28):11470–5. Epub 2011/06/29. 1103873108 [pii] 10.1073/pnas.1103873108 21709251 PMC3136256

[17] Kim J, Frey WD, He H, Kim H, Ekram MB, Bakshi A, et al Peg3 mutational effects on reproduction and placenta-specific gene families. PLoS One. 2013;8(12):e83359 Epub 2014/01/07. 10.1371/journal.pone.0083359 PONE-D-13-37965 [pii]. 24391757 PMC3877027

[18] Chiavegatto S, Sauce B, Ambar G, Cheverud JM, Peripato AC. Hypothalamic expression of Peg3 gene is associated with maternal care differences between SM/J and LG/J mouse strains. Brain Behav. 2012;2(4):365–76. Epub 2012/09/06. 10.1002/brb3.58 22950040 PMC3432959

[19] Moore T, Haig D. Genomic imprinting in mammalian development: a parental tug-of-war. Trends Genet. 1991;7(2):45–9. Epub 1991/02/01. 0168-9525(91)90230-N [pii] 10.1016/0168-9525(91)90230-N . 2035190

[20] Keverne EB, Curley JP. Epigenetics, brain evolution and behaviour. Front Neuroendocrinol. 2008;29(3):398–412. Epub 2008/04/29. S0091-3022(08)00007-1 [pii] 10.1016/j.yfrne.2008.03.001 . 18439660

[21] Mitchell KJ, Pannerec A, Cadot B, Parlakian A, Besson V, Gomes ER, et al Identification and characterization of a non-satellite cell muscle resident progenitor during postnatal development. Nat Cell Biol. 2010;12(3):257–66. Epub 2010/02/02. ncb2025 [pii] 10.1038/ncb2025 . 20118923

[22] Pannerec A, Formicola L, Besson V, Marazzi G, Sassoon DA. Defining skeletal muscle resident progenitors and their cell fate potentials. Development. 2013;140(14):2879–91. Epub 2013/06/07. dev.089326 [pii] 10.1242/dev.089326 . 23739133

[23] Bonfanti C, Rossi G, Tedesco FS, Giannotta M, Benedetti S, Tonlorenzi R, et al PW1/Peg3 expression regulates key properties that determine mesoangioblast stem cell competence. Nat Commun. 2015;6:6364 Epub 2015/03/10. ncomms7364 [pii] 10.1038/ncomms7364 . 25751651 PMC4366533

[24] Relaix F, Wei XJ, Wu X, Sassoon DA. Peg3/Pw1 is an imprinted gene involved in the TNF-NFkappaB signal transduction pathway. Nat Genet. 1998;18(3):287–91. Epub 1998/03/21. 10.1038/ng0398-287 . 9500555

[25] Relaix F, Wei X, Li W, Pan J, Lin Y, Bowtell DD, et al Pw1/Peg3 is a potential cell death mediator and cooperates with Siah1a in p53-mediated apoptosis. Proc Natl Acad Sci U S A. 2000;97(5):2105–10. Epub 2000/02/19. 10.1073/pnas.040378897 040378897 [pii]. 10681424 PMC15761

[26] Jiang X, Yu Y, Yang HW, Agar NY, Frado L, Johnson MD. The imprinted gene PEG3 inhibits Wnt signaling and regulates glioma growth. J Biol Chem. 2010;285(11):8472–80. Epub 2010/01/13. M109.069450 [pii] 10.1074/jbc.M109.069450 20064927 PMC2832996

[27] Thiaville MM, Huang JM, Kim H, Ekram MB, Roh TY, Kim J. DNA-binding motif and target genes of the imprinted transcription factor PEG3. Gene. 2013;512(2):314–20. Epub 2012/10/20. S0378-1119(12)01254-1 [pii] 10.1016/j.gene.2012.10.005 23078764 PMC3513644

[28] Perera BP, Teruyama R, Kim J. Yy1 gene dosage effect and bi-allelic expression of peg3. PLoS One. 2015;10(3):e0119493 Epub 2015/03/17. 10.1371/journal.pone.0119493 PONE-D-14-41735 [pii]. 25774914 PMC4361396

[29] Rodriguez CI, Buchholz F, Galloway J, Sequerra R, Kasper J, Ayala R, et al High-efficiency deleter mice show that FLPe is an alternative to Cre-loxP. Nat Genet. 2000;25(2):139–40. Epub 2000/06/03. 10.1038/75973 . 10835623

[30] Lallemand Y, Luria V, Haffner-Krausz R, Lonai P. Maternally expressed PGK-Cre transgene as a tool for early and uniform activation of the Cre site-specific recombinase. Transgenic Res. 1998;7(2):105–12. Epub 1998/06/03. . 9608738 10.1023/a:1008868325009

[31] Caldwell JD, Greer ER, Johnson MF, Prange AJ Jr., Pedersen CA. Oxytocin and vasopressin immunoreactivity in hypothalamic and extrahypothalamic sites in late pregnant and postpartum rats. Neuroendocrinology. 1987;46(1):39–47. Epub 1987/06/01. . 3614554 10.1159/000124794

[32] Pedersen CA, Prange AJ Jr. Induction of maternal behavior in virgin rats after intracerebroventricular administration of oxytocin. Proc Natl Acad Sci U S A. 1979;76(12):6661–5. Epub 1979/12/01. 293752 10.1073/pnas.76.12.6661 PMC411928

[33] Donaldson ZR, Young LJ. Oxytocin, vasopressin, and the neurogenetics of sociality. Science. 2008;322(5903):900–4. Epub 2008/11/08. 322/5903/900 [pii] 10.1126/science.1158668 . 18988842

[34] Young LJ, Winslow JT, Wang Z, Gingrich B, Guo Q, Matzuk MM, et al Gene targeting approaches to neuroendocrinology: oxytocin, maternal behavior, and affiliation. Horm Behav. 1997;31(3):221–31. Epub 1997/06/01. S0018-506X(97)91377-5 [pii] 10.1006/hbeh.1997.1377 . 9213136

[35] Nishimori K, Young LJ, Guo Q, Wang Z, Insel TR, Matzuk MM. Oxytocin is required for nursing but is not essential for parturition or reproductive behavior. Proc Natl Acad Sci U S A. 1996;93(21):11699–704. Epub 1996/10/15. 8876199 10.1073/pnas.93.21.11699 PMC38121

[36] Curley JP, Barton S, Surani A, Keverne EB. Coadaptation in mother and infant regulated by a paternally expressed imprinted gene. Proc Biol Sci. 2004;271(1545):1303–9. Epub 2004/08/13. 10.1098/rspb.2004.2725 6A65N9DT2AP4W9UJ [pii]. 15306355 PMC1691726

[37] Frey WD, Kim J. Tissue-Specific Contributions of Paternally Expressed Gene 3 in Lactation and Maternal Care of Mus musculus. PLoS One. 2015;10(12):e0144459 Epub 2015/12/08. 10.1371/journal.pone.0144459 PONE-D-15-35159 [pii]. 26640945 PMC4671625

[38] Champagne FA, Curley JP, Swaney WT, Hasen NS, Keverne EB. Paternal influence on female behavior: the role of Peg3 in exploration, olfaction, and neuroendocrine regulation of maternal behavior of female mice. Behav Neurosci. 2009;123(3):469–80. Epub 2009/06/03. 2009-07961-001 [pii] 10.1037/a0015060 . 19485553

[39] Olson EN, Arnold HH, Rigby PW, Wold BJ. Know your neighbors: three phenotypes in null mutants of the myogenic bHLH gene MRF4. Cell. 1996;85(1):1–4. Epub 1996/04/05. S0092-8674(00)81073-9 [pii]. . 8620528 10.1016/s0092-8674(00)81073-9

[40] Zakany J, Gerard M, Favier B, Duboule D. Deletion of a HoxD enhancer induces transcriptional heterochrony leading to transposition of the sacrum. EMBO J. 1997;16(14):4393–402. Epub 1997/07/16. 10.1093/emboj/16.14.4393 9250683 PMC1170065

[41] Gerard M, Hernandez L, Wevrick R, Stewart CL. Disruption of the mouse necdin gene results in early post-natal lethality. Nat Genet. 1999;23(2):199–202. Epub 1999/10/03. 10.1038/13828 . 10508517

[42] Glasgow E, Ryu SL, Yamashita M, Zhang BJ, Mutsuga N, Gainer H. APeg3, a novel paternally expressed gene 3 antisense RNA transcript specifically expressed in vasopressinergic magnocellular neurons in the rat supraoptic nucleus. Brain Res Mol Brain Res. 2005;137(1–2):143–51. Epub 2005/06/14. S0169-328X(05)00108-7 [pii] 10.1016/j.molbrainres.2005.02.030 . 15950772

[43] Choo JH, Kim JD, Kim J. Imprinting of an evolutionarily conserved antisense transcript gene APeg3. Gene. 2008;409(1–2):28–33. Epub 2008/01/02. S0378-1119(07)00565-3 [pii] 10.1016/j.gene.2007.10.036 18166281 PMC2259222

[44] Frey WD, Kim J. APeg3: regulation of Peg3 through an evolutionarily conserved ncRNA. Gene. 2014;540(2):251–7. Epub 2014/03/04. S0378-1119(14)00258-3 [pii] 10.1016/j.gene.2014.02.056 24582979 PMC4028329

[45] Yang M, Weber MD, Crawley JN. Light phase testing of social behaviors: not a problem. Front Neurosci. 2008;2(2):186–91. Epub 2009/02/20. 10.3389/neuro.01.029.2008 19225591 PMC2622744

[46] Broad KD, Keverne EB. Placental protection of the fetal brain during short-term food deprivation. Proc Natl Acad Sci U S A. 2011;108(37):15237–41. Epub 2011/08/04. 1106022108 [pii] 10.1073/pnas.1106022108 21810990 PMC3174621

[47] Wolf JB, Hager R. A maternal-offspring coadaptation theory for the evolution of genomic imprinting. PLoS Biol. 2006;4(12):e380 Epub 2006/11/16. 06-PLBI-RA-1349R2 [pii] 10.1371/journal.pbio.0040380 17105351 PMC1635750

[48] Cowley M, Garfield AS, Madon-Simon M, Charalambous M, Clarkson RW, Smalley MJ, et al Developmental programming mediated by complementary roles of imprinted Grb10 in mother and pup. PLoS Biol. 2014;12(2):e1001799 Epub 2014/03/04. 10.1371/journal.pbio.1001799 PBIOLOGY-D-13-02196 [pii]. 24586114 PMC3934836

[49] Wolf JB, Cowley M, Ward A. Coadaptation between mother and offspring: why not? PLoS Biol. 2015;13(3):e1002085 Epub 2015/03/19. 10.1371/journal.pbio.1002085 PBIOLOGY-D-14-03934 [pii]. 25786111 PMC4365009

[50] Ye A, He H, Kim J. Paternally expressed Peg3 controls maternally expressed Zim1 as a trans factor. PLoS One. 2014;9(9):e108596 Epub 2014/09/30. 10.1371/journal.pone.0108596 PONE-D-14-21806 [pii]. 25265264 PMC4180786

[51] Radford EJ, Isganaitis E, Jimenez-Chillaron J, Schroeder J, Molla M, Andrews S, et al An unbiased assessment of the role of imprinted genes in an intergenerational model of developmental programming. PLoS Genet. 2012;8(4):e1002605 Epub 2012/04/19. 10.1371/journal.pgen.1002605 PGENETICS-D-11-01881 [pii]. 22511876 PMC3325178

