# Supplementary figures and images for: A Novel Mutant Allele of Pw1/Peg3 Does Not Affect Maternal Behavior or Nursing Behavior
Source: PLoS Genet. 2016 May 17;12(5):e1006053. doi: 10.1371/journal.pgen.1006053 (PMC4871489; doi:10.1371/journal.pgen.1006053)

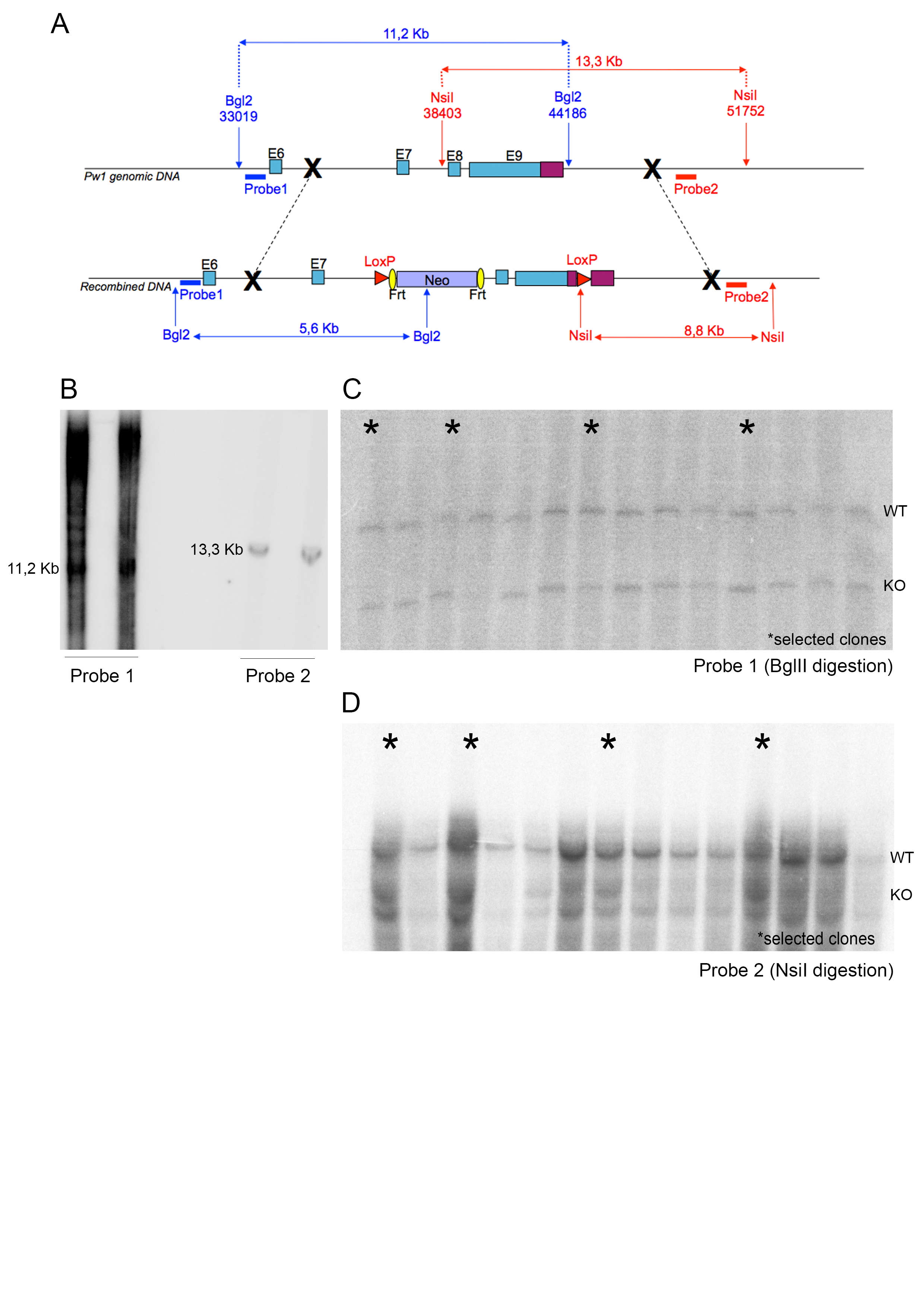

Supplement: S1 Fig — A. Scheme representing probes 1 and 2 used for the Southern Blot analyses. Black crosses indicate recombination sites. Proper insertion is predicted to generate distinct genomic fragments due to the presence of BglII (Bgl2) and NsiI restriction sites. Specifically, a 5,6 Kb and a 8,8 Kb fragment are detected with probes 1 and 2 respectively in the recombined (KO) allele whereas a 11,2 Kb and a 13,3 Kb fragment are predicted for the wildtype (WT) allele. B. Prior to Southern on the ES clones, probes 1 and 2 were tested in duplicate by Southern on C57Bl6 genomic DNA digested by BglII and NsiI respectively. C-D. Southern Blot on ES cells clones using probe 1 (C) and probe 2 (D). Out of ~600 Neomycin-selected-ES cells clones, 14 clones were identified by probes 1 and 2. Out of those 14, four clones (*) were finally selected for the blastocysts injection. (TIF) [file pgen.1006053.s002.tif]

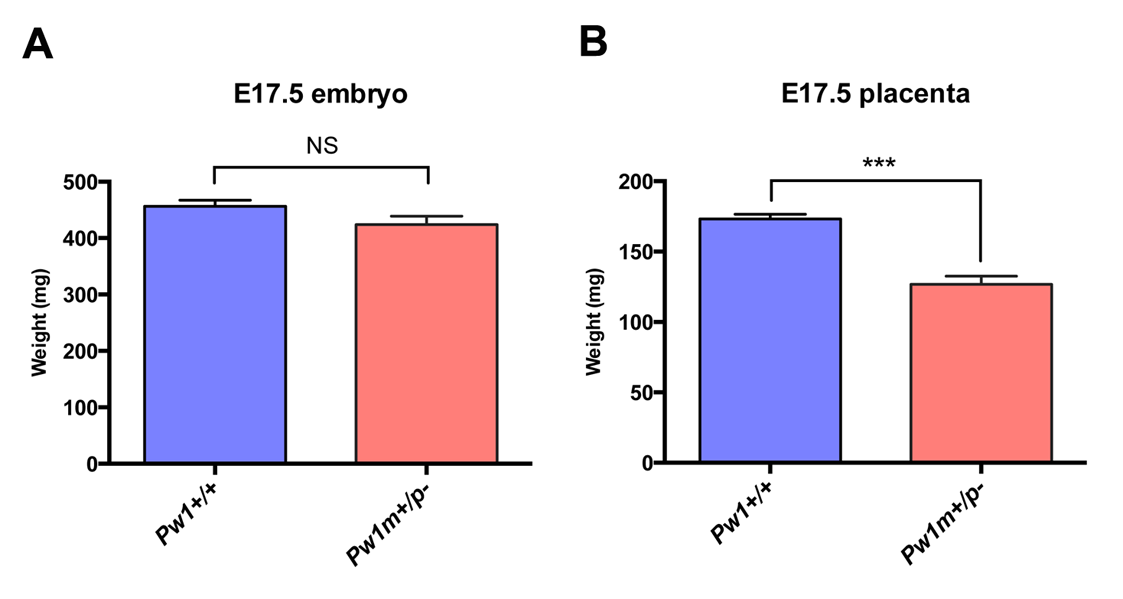

Supplement: S2 Fig — A. Weight of Pw1+/+ compared to Pw1m+/p- E17.5 littermate embryos. B. Weight of Pw1+/+ compared to Pw1m+/p- E17.5 littermate embryos' placenta. Panel A. Pup retrieval latency: Values were not all normally distributed (P ≥ 0.0041, D'Agostino and Pearson omnibus normality test). Using Kruskal-Wallis test (P = 0.4171) no significant differences were found between all four genotypes. Nest building latency: Values were not all normally distributed (P ≥ 0.0383, D'Agostino and Pearson omnibus normality test). Using Kruskal-Wallis test (P = 0.1032) no significant differences were found between all four genotypes. Nest quality: Values were all normally distributed (P > 0.2142, D'Agostino and Pearson omnibus normality test). Using ordinary one-way ANOVA (F3,46 = 1.954; P = 0.1341) no significant differences were found between all four genotypes. Time spent crouching: Values were all normally distributed (P > 0.1567, D'Agostino and Pearson omnibus normality test). Using ordinary one-way ANOVA (F3,46 = 0.7315; P = 0.5385) no significant differences were found between all four genotypes. Pup sniffing latency: Values were not all normally distributed (P ≥ 0.0011, D'Agostino and Pearson omnibus normality test). Using Kruskal-Wallis test (P = 0.3885) no significant differences were found between all four genotypes. Panel B: Pup retrieval latency: Values were not all normally distributed (P < 0.0001, D'Agostino and Pearson omnibus normality test). Using Kruskal-Wallis test (P = 0.1346) no significant differences were found between all four genotypes. Nest building latency: Values were all normally distributed (P > 0.5123, D'Agostino and Pearson omnibus normality test). Using ordinary one-way ANOVA (F3,48 = 0.8627; P = 0.4669) no significant differences were found between all four genotypes. Nest quality: Values were not all normally distributed (P < 0.0001, D'Agostino and Pearson omnibus normality test). Using Kruskal-Wallis test (P = 0.5854) no significant differences were found be [file pgen.1006053.s003.tif]

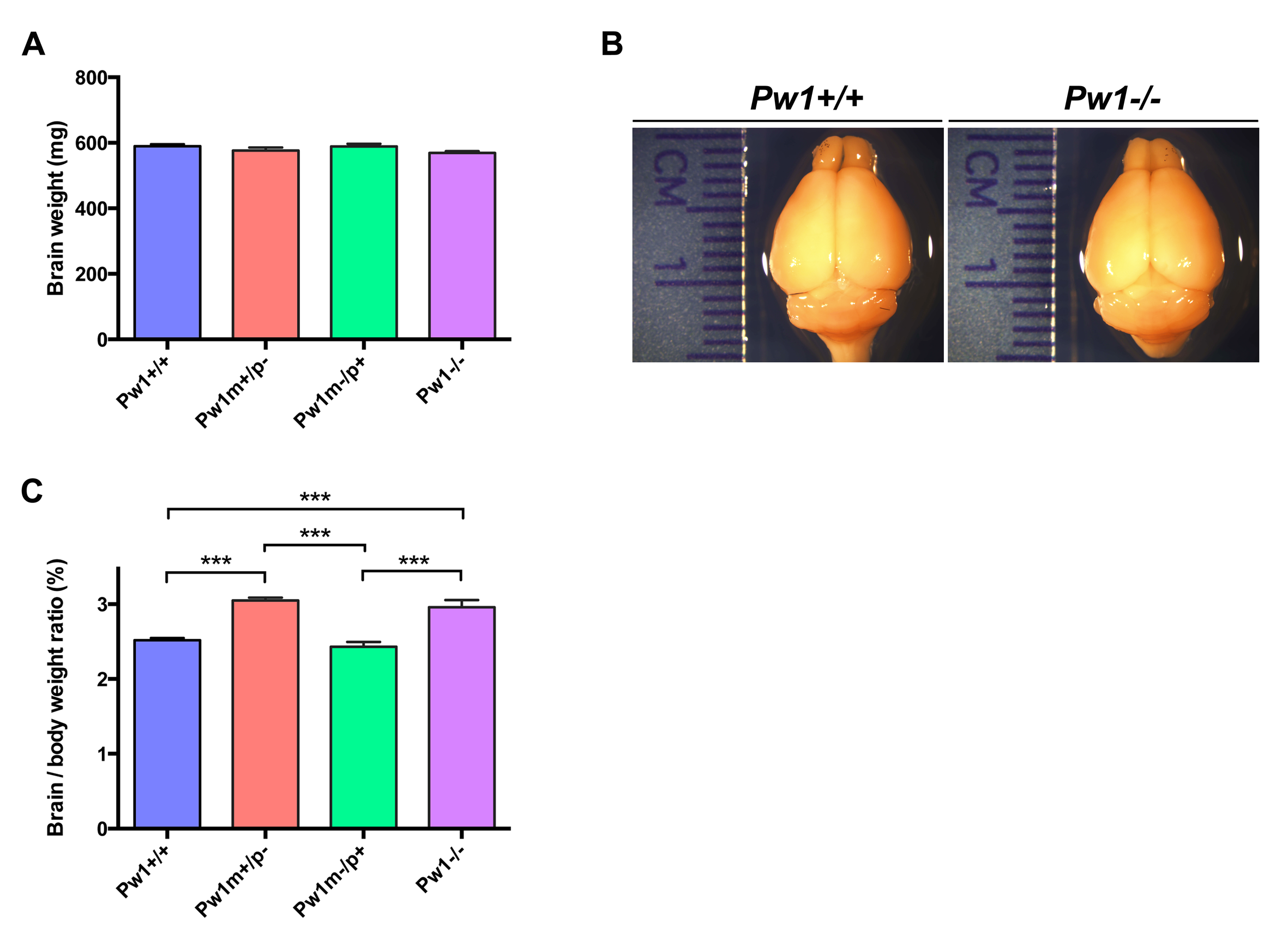

Supplement: S3 Fig — A. Brain weights of 2 months old Pw1+/+, Pw1m+/p-, Pw1m-/p+ and Pw1-/- males. B. Representative picture of 2 months old Pw1+/+ and Pw1-/- brains. C. Brain over body weights ratio of 2 months old Pw1+/+, Pw1m+/p-, Pw1m-/p+ and Pw1-/- males. In all graphs, values represent mean ± s.e.m. Statistical analysis was performed using one-way ANOVA (n≥4). *P<0.05, **P<0.01 and ***P<0.001. D. Upper panel—PVN. All values were normally distributed (P > 0.1000, Kolmogorov-Smirnov test). Using one-way ANOVA (F3,21 = 2.468; P = 0.0902) no significant differences were found between all four genotypes. Middle panel—SON. All values were normally distributed (P > 0.0848, Kolmogorov-Smirnov test). Using one-way ANOVA (F3,20 = 0.07157; P = 0.9745) no significant differences were found between all four genotypes. Bottom panel—MPOA. All values were normally distributed (P > 0.1000, Kolmogorov-Smirnov test). Using unpaired t test no significant differences were found between the two genotypes (P = 05924). F. Using two-way ANOVA (Interaction: F3,57 = 0.7796; P = 0.5102) no significant differences were found. (TIF) [file pgen.1006053.s004.tif]

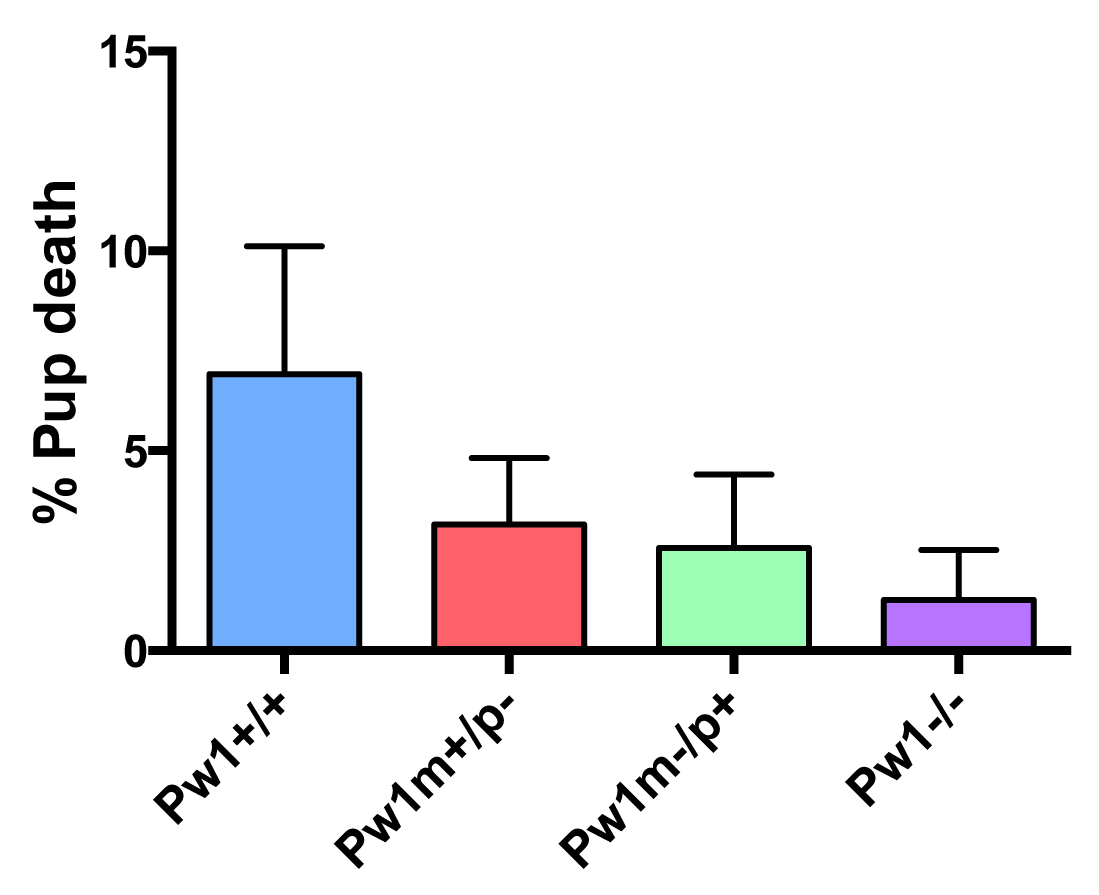

Supplement: S4 Fig — Values represent mean ± s.e.m. Statistical analysis was performed using one-way ANOVA Kruskal-Wallis test (n≥12 litters). (TIF) [file pgen.1006053.s005.tif]

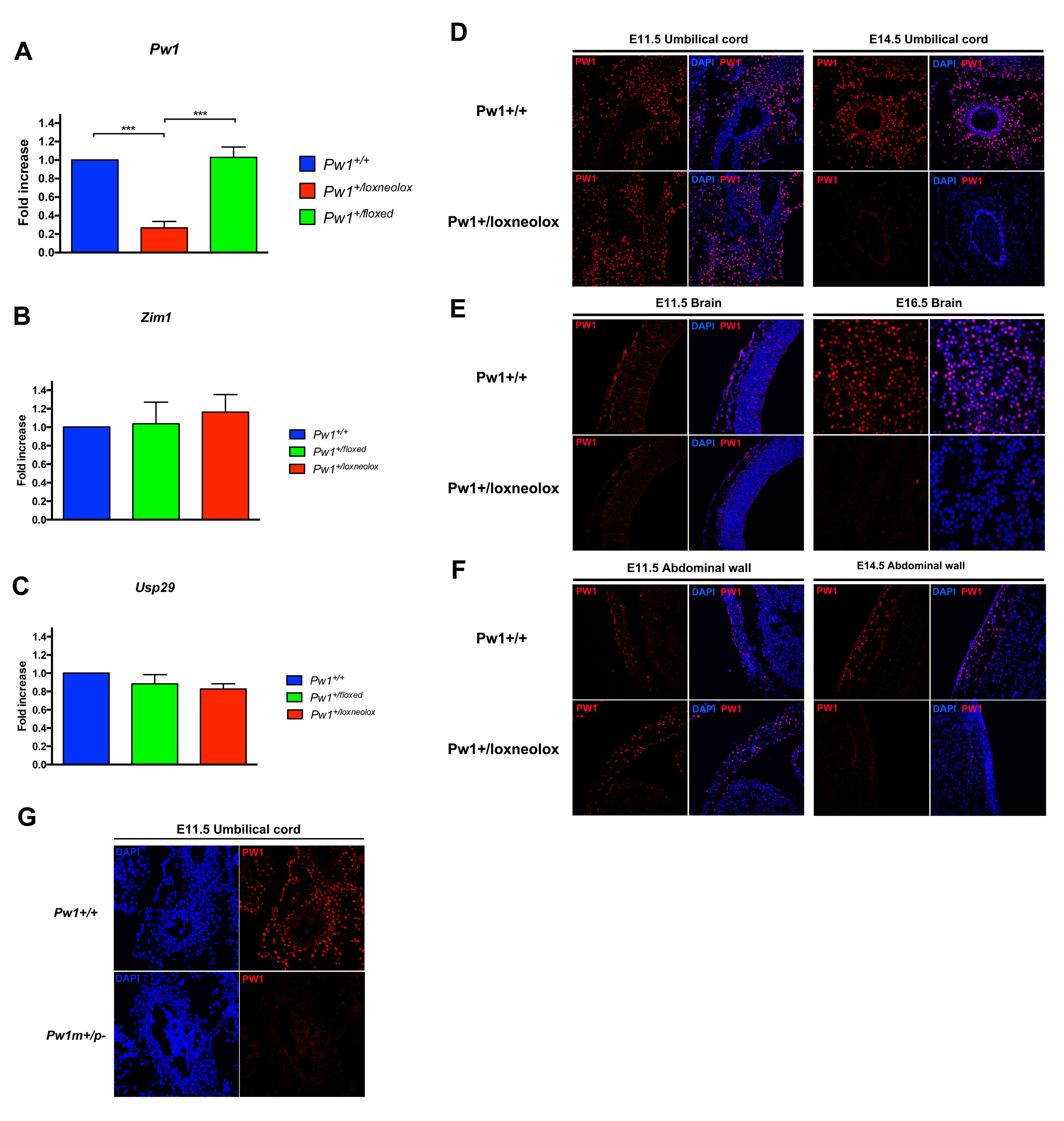

Supplement: S5 Fig — A-C. Postnatal day 0 brains were analyzed for Pw1 (A), Zim1 (B), and Usp29 (C) expression levels by RT-qPCR. Values represent fold increase ± s.e.m. normalized to Hprt1 expression level. Statistical analysis was performed using one-way ANOVA Kruskal-Wallis test (n = 3). *P<0.05, **P<0.01 and ***P<0.001. D-F. Immunostaining for PW1 on E11.5, E14.5, and E16.5 Pw1+/+ and Pw1+/loxneolox embryos (8μm thick sections). D. Umbilical cord. E. Brain. F. Abdominal wall. In Pw1+/loxneolox embryos, neomycin insertion irreversibly shuts down PW1 expression between E11.5 and E14.5. G. Immunostaining for PW1 on E11.5 Pw1+/+ and Pw1m+/p- embryos (8μm thick sections). As expected, paternal loss of Pw1 abrogates PW1 expression. Here is shown the umbilical cord, a strong site of PW1 expression at E11.5. (TIF) [file pgen.1006053.s006.tif]

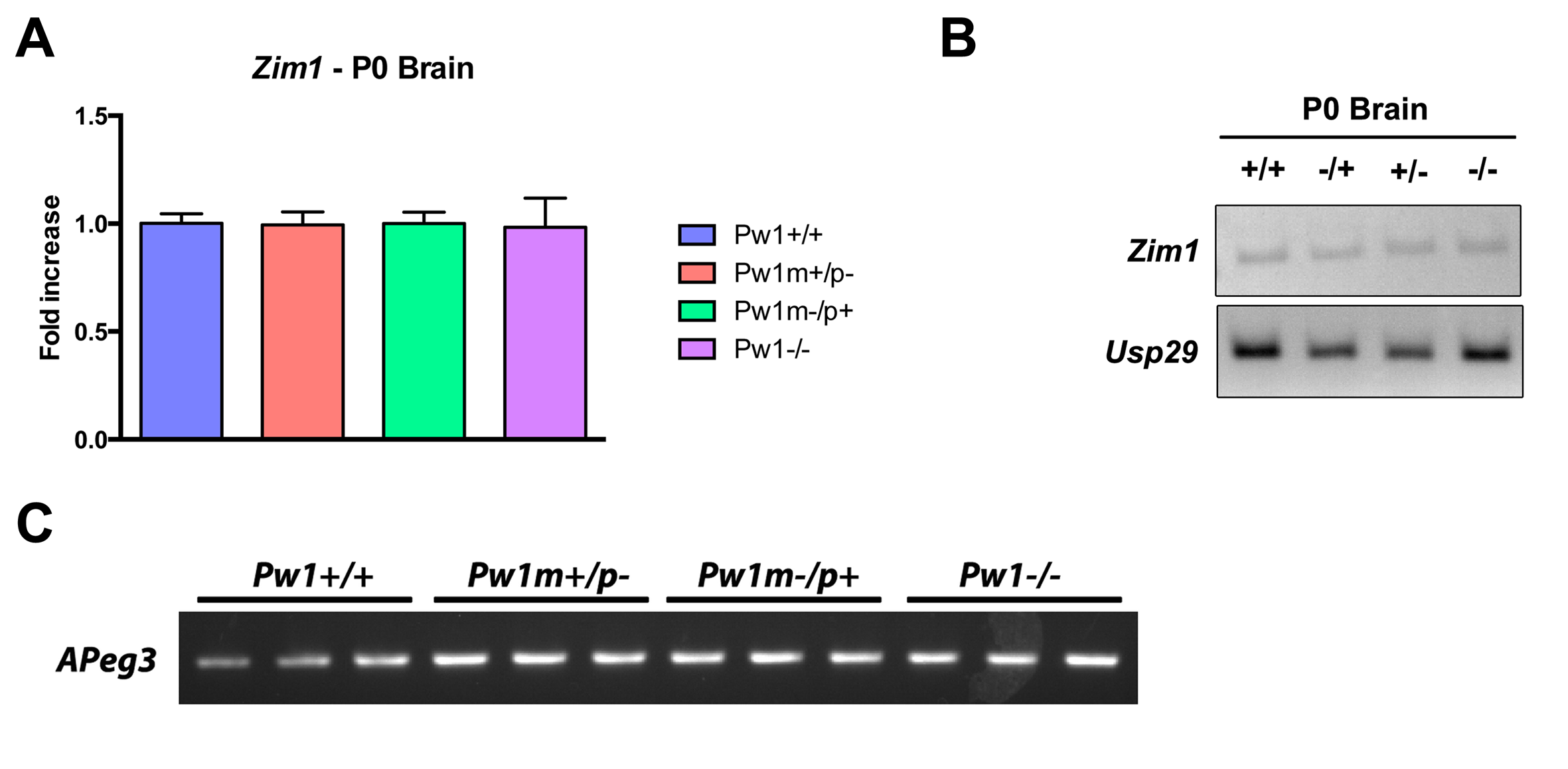

Supplement: S6 Fig — A. Zim1 real time RT-PCR on postnatal day 0 brains (n = 3 per genotype). Gene expression levels are normalized using Hprt1 gene expression. B. Zim1 and Usp29 transcript levels analyzed by semi-quantitative RT-PCR on postnatal day 0 brains (n = 3 per genotype). C. APeg3 transcript level analysis on postnatal day 0 brains using First-Strand RT-PCR. Each well corresponds to a different sample. (TIF) [file pgen.1006053.s007.tif]
